# Tissue growth constrains root organ outlines into an isometrically scalable shape

**DOI:** 10.1242/dev.196253

**Published:** 2021-02-26

**Authors:** Motohiro Fujiwara, Tatsuaki Goh, Satoru Tsugawa, Keiji Nakajima, Hidehiro Fukaki, Koichi Fujimoto

**Affiliations:** 1 Department of Biological Sciences, Graduate School of Science, Osaka University, Machikaneyama-cho, Toyonaka 560-0043, Japan; 2 Graduate School of Science and Technology, Nara Institute of Science and Technology, Takayama, Ikoma 630-0192, Japan; 3 Department of Biology, Graduate School of Science, Kobe University, Rokkodai, Kobe 657-8501, Japan

**Keywords:** Catenary curve, Organ shape, Plant root tip, Scaling, Anisotropic growth, Developmental constraint

## Abstract

Organ morphologies are diverse but also conserved under shared developmental constraints among species. Any geometrical similarities in the shape behind diversity and the underlying developmental constraints remain unclear. Plant root tip outlines commonly exhibit a dome shape, which likely performs physiological functions, despite the diversity in size and cellular organization among distinct root classes and/or species. We carried out morphometric analysis of the primary roots of ten angiosperm species and of the lateral roots (LRs) of *Arabidopsis*, and found that each root outline was isometrically scaled onto a parameter-free catenary curve, a stable structure adopted for arch bridges. Using the physical model for bridges, we analogized that localized and spatially uniform occurrence of oriented cell division and expansion force the LR primordia (LRP) tip to form a catenary curve. These growth rules for the catenary curve were verified by tissue growth simulation of developing LRP development based on time-lapse imaging. Consistently, LRP outlines of mutants compromised in these rules were found to deviate from catenary curves. Our analyses demonstrate that physics-inspired growth rules constrain plant root tips to form isometrically scalable catenary curves.

## Abstract (precis)

**Highlighted Article:** The dome-shaped outlines of plant root tips converge to a parameter-free catenary curve seen in arch bridges, owing to a constraint from anisotropic and localized tissue growth.

## INTRODUCTION

Plant and animal organ forms (i.e. outline morphologies of organs) are defined by size and shape. Organ forms became diverse across species as a consequence of adaptation to various physiological and environmental conditions during evolutionary radiation (Darwin, 1859; Le Roy et al., 2019; Maugarny-Cales and Laufs, 2018; Salcedo et al., 2019; Tsukaya, 2018). However, organ forms share a conservative feature in each organ type (e.g. roots and leaves in plants, and beaks and wings in animals) (Houle et al., 2017; Wang and Clarke, 2015). As a typical example of similarity behind the diversity, organ outlines can collapse onto a single common shape across species by rescaling of individual size [e.g. cannon bones of ox, sheep and giraffe (Thompson, 1917), and beaks of songbirds (Abzhanov, 2017; Campas et al., 2010)]. The scaling of songbird beaks is imposed by developmental programs shared among species (Abzhanov, 2017; Campas et al., 2010; Fritz et al., 2014). Although the scaling of organ size (e.g. proportionality and allometry to body size) has been extensively studied (Niklas, 1994; Schmidt-Nielsen, 1984), quantitative assessments on the shape scaling and underlying developmental constraints have only been reported in a limited number of cases, as mentioned above.

**Fig. 1.**
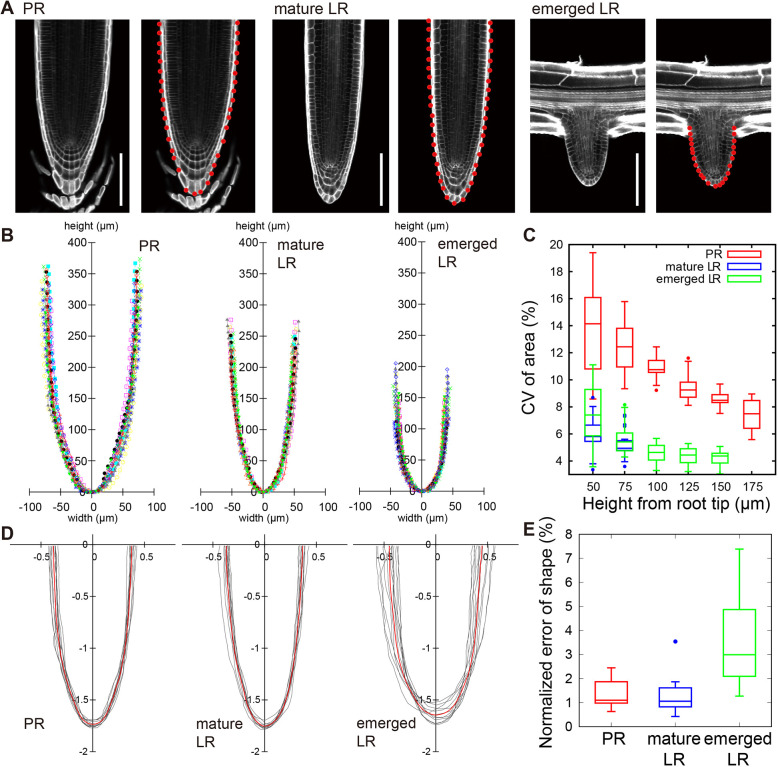
**Reproducible size and shape of root tip outlines in *Arabidopsis*.** (A) Longitudinal confocal sections of a PR, a mature LR and an emerged LR. Cell walls were stained with SR2200. Red points indicate cell junctions on the dome outline. Scale bars: 100 µm. (B) Reproducibility of root tip size. Outlines of multiple samples from each root class were superimposed with different colors. Points indicate cell junctions on the outline. (C) Reproducibility of root tip area. Root tip areas measured on the median longitudinal section up to the indicated heights from the root tip. Size reproducibility is indicated by CV [CV (%)= (s.d. of area)×100/(mean of area)]. Higher CV of PR than that of LR is likely attributable to phase differences of root cap sloughing among samples. (D) Reproducibility of root tip shape. Outlines of multiple root samples were normalized by the radial Fourier series expansion method (Materials and Methods) and superimposed (gray). Median outlines are shown in red. (E) A graph showing the shape reproducibility indicator (Eqn 9) of tip outlines for distinct root types. The upper and lower hinges, the middle lines and the error bars of the box plots in C and E represent the 25th, 75th and 50th (median) percentiles, and s.d., respectively. B and E are drawn from identical data sets [*n*=12 (PR), *n*=12 (mature LR) and *n*=11 (emerged LR)].

The outline of plant root tips commonly exhibits a domed shape in angiosperms, despite diversities in size and cellular organization among species and/or in developmental processes among root classes (Clowes, 2000; Hamamoto et al., 2006; Heimsch and Seago, 2008). The root tip plays a pivotal role in root growth by executing a wide variety of functions, such as penetration, anchorage, gravity perception, and nutrient and water uptake (Eshel and Beeckman, 2013). The root tip mainly consists of the root apical meristem (RAM) and the surrounding root cap (Fig. S1A-C) (Kumpf and Nowack, 2015; Petricka et al., 2012). RAM organization is diverse across species, as exemplified by open and closed meristems (Clowes, 2000; Heimsch and Seago, 2008), and the number of cell files and layers (Di Ruocco et al., 2018; Hamamoto et al., 2006; Mellor et al., 2019). Even within a given individual, there are several classes of developmentally distinct roots, such as primary roots (PRs), lateral roots (LRs) and adventitious roots (ARs). PRs are established during embryogenesis (Petricka et al., 2012; ten Hove et al., 2015), whereas LRs and ARs are post-embryonically initiated in existing roots and specific parts of the shoot, respectively (Fig. 1A; Lavenus et al., 2013). Although internal morphologies of PRs and LRs have been extensively studied at the level of cellular organization and shown to be largely conserved (Petricka et al., 2012), how their outline morphologies have converged into a seemingly similar dome shape and whether any mechanical impositions play a role to stabilize fixed root tip shapes, if any, remain unknown.

LR primordia (LRP) development is a suitable model system to investigate the nature of tissue growth that governs the formation and maintenance of the root tip outlines (Goh et al., 2016; von Wangenheim et al., 2016). In *Arabidopsis thaliana*, LRP originates from the LR founder cells that are specified in the xylem pole pericycle in the differentiation zone (Lavenus et al., 2013; Norman et al., 2013). LR founder cells undergo multiple rounds of coordinated cell divisions and expansion to produce a dome-shaped LRP that emerges from the overlaying tissues to extend into the soil (Goh et al., 2016; von Wangenheim et al., 2016). Several factors, such as cell division rules arising from cell geometry and mechanical constraints by the overlaying tissues, have been reported to affect the LRP outline (Lucas et al., 2013; Vermeer et al., 2014; von Wangenheim et al., 2016).

Here, we performed morphometric analysis of the PR and LR tip outlines and revealed that they are highly reproducible in both size and shape within a given species and regardless of the root class. Statistical analyses showed that the outlines of different root classes and species were isometrically scalable (geometrically similar); by scaling the width and the height of root tips with an identical rate, the outlines commonly converge to a unique catenary curve. Simulations incorporating cell division and expansion rules drawn from time-lapse observation of LRP development identified tissue growth constraints as a major determinant for the geometry and mechanics of the isometrically-scalable root tip shape. The developmental constraints identified in this study govern the scalable diversity of root organ morphologies.

## RESULTS

### Reproducible size and shape of the root tip dome in *Arabidopsis*

PR and LR tips of *Arabidopsis* share an apparently common dome shape with a nearly perfect rotational symmetry (Fig. 1A; Fig. S1A). In order to quantitatively evaluate the shape of *Arabidopsis* root tips, we captured longitudinal optical sections of the tips of PRs, mature LRs (longer than 5 mm as measured from the primary root surface) and young emerged LRs (less than 200 µm as measured above; Fig. 1A; Materials and Methods). Root tip outlines were delineated semi-automatically by marking the positions of cell-cell junctions along the outer surface of the outermost cell layer (red points in the right panels of each root class in Fig. 1A), and then projected to the spatial coordinate (x, y) (Fig. S1D-G; Materials and Methods). The size and shape of the extracted outlines were apparently reproducible within each root class (Fig. 1B). First, we quantified the size reproducibility based on the coefficient of variation (CV, i.e. the s.d. divided by the mean). The CV was within a range of a few percent for LRs (4-7% for dome area, Fig. 1C; and 3-6% for dome width, Fig. S1G, Fig. S2), but was slightly larger for PRs (7-14% for dome area, Fig. 1C; 5-11% for dome width, Fig. S2). We then assessed shape reproducibility irrespective of the size by normalizing the root tip outlines (Hervieux et al., 2017; Hong et al., 2016). An indicator of shape reproducibility, which was represented by the root mean squared error (MSE) between the normalized outlines of individual root tips and their average (Eqn. 9 in Materials and Methods; Fig. 1D), was found within a range of 1-3% (Fig. 1E). Taken together, our analysis indicated that *Arabidopsis* PR and LR tips are highly reproducible in both size and shape.

### Tip dome outlines of PRs and LRs fit to a catenary curve and its essentially-equivalent curve, a catenary-closest ellipse

**Fig. 2.**
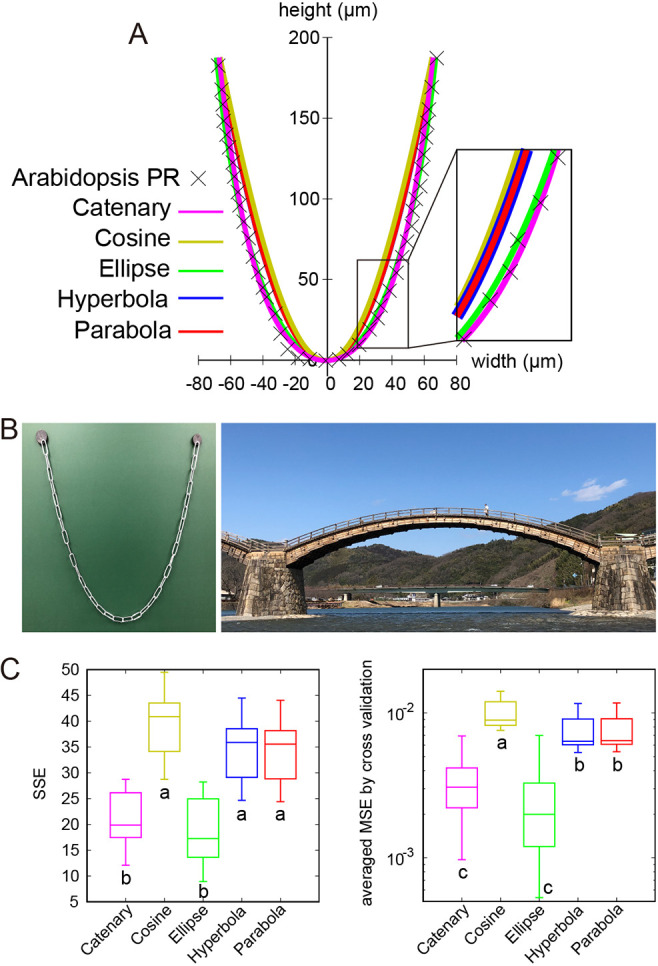
**Catenary is an isometrically scalable function and the best-fit model for root tip outlines.** (A) NLS fitting of a representative *Arabidopsis* PR outline with five geometrical functions (catenary, cosine, ellipse, hyperbola and parabola). (B) Examples of catenary curves in architectures: a chain hanging with its both ends fixed under gravity (left panel) and the Kintai wooden bridge in Yamaguchi prefecture, Japan (right panel). (C) SSE between PR sample dome outlines (*n*=12) and the indicated curve function (left panel). The averaged MSE by cross validation between PR sample dome outlines (*n*=12) and the indicated curve function (right panel; Eqn 12). Different letters (a, b, c) denote statistically significant differences (*P*<0.05) among means by Tukey's honestly significant difference test. The upper and lower hinges, the middle lines and the error bars of box plots represent the 25th, 75th, and 50th percentiles, and s.d., respectively.

The reproducibility of root outline shapes (Fig. 1) prompted us to examine which mathematical function accurately represents the dome shape. We assessed which of the five representative curves – an ellipse, parabola, hyperbola, cosine or catenary – best fits the root tip outlines (Fig. 2A; Materials and Methods for statistical analysis). Although the outlines of RAM and shoot apical meristem have been previously fitted to an ellipse (Colombi et al., 2017) and to a parabola (Leiboff et al., 2016, 2015), respectively, whether these outlines could be better fitted to other dome-shaped functions with a common mathematical nature (hyperbola) or a mechanical stability [cosine (Timoshenko and Gere, 1961) and catenary (Block et al., 2006; Lockwood, 1961) (Fig. 2B)] has not been investigated. The ellipse and catenary functions were found to fit equally well to the outline data of PRs, emerged LRs and mature LRs of *Arabidopsis*, and fit significantly better than the other three functions [the sample standard error (SSE) in Fig. 2C (left panel) and Fig. S3 (left panels); averaged MSE of cross validation in Fig. 2C (right panels) and Fig. S3 (right panels); and the Akaike information criterion (AIC) in Fig. S3 (middle panels)]. Interestingly, the fitted ellipse and catenary functions were found to superimpose each other within the range of the root tip width (Fig. 2A). Indeed, this characteristic ellipse had the highest similarity to the catenary curve among any ellipses at a level that could be nearly identical in shape (Fig. S4A-J), and is therefore hereafter referred to as a catenary-closest ellipse. This indicates that a catenary curve and a catenary-closest ellipse are essentially equivalent functions best fitting to the root tip outlines of both *Arabidopsis* PR and LR.

### Isometric scaling unifies dome outlines of the root tips of diverse root classes and plant species into a single common shape

**Fig. 3.**
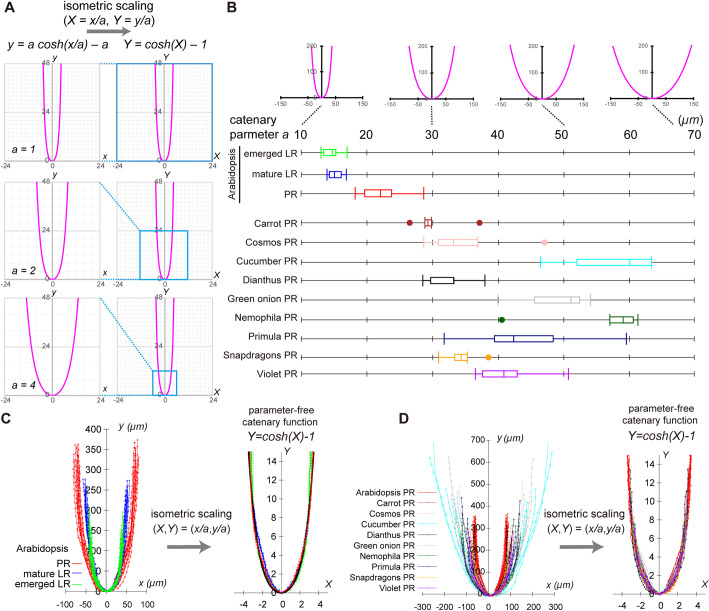
**Isometrically scalable root tip outlines to a parameter-free catenary curve.** (A) Isometric scalability of catenary function. Catenary curves [*y=a* cosh*(x/a)* – *a*] with *a=*1, 2 and 4 (left panels) are isometrically scalable into a parameter-free catenary function [*Y=*cosh(*X*) − 1, *X*=*x/a*, *Y*=*y/a*, right panels]. (B) Catenary curves with *a=*10, 30, 50 and 70 (upper panel). Catenary parameter *a* of root tip outlines quantified by the NLS method (bottom panel). *Arabidopsis* PR, mature and emerged LR outlines [*n*=12 (PR), *n*=12 (mature LR) and *n*=11 (emerged LR)], and PR of nine angiosperm species (*n*=5 for each species) were analyzed. The fitted value of *a* indicated high reproducibility in *Arabidopsis* (CV of *a* ∼14% in PR, ∼7.2% in mature LR and ∼8.5% in emerged LR), consistently with the level of size reproducibility (CV of root tip area in Fig. 1C), and was, on average, 50% larger in the PR than in the LR. The right and left hinges, the middle lines and the error bars of box plots represent the 25th, 75th and 50th percentiles, and s.d., respectively. (C,D) Outlines of *Arabidopsis* PRs and LRs (left panel in C), and the PRs of ten angiosperm species (left panel in D) were isometrically scalable to a parameter-free catenary curve using distinct catenary parameter *a* (respective right panels). Samples in C and D are identical data sets to B. Sample sets of *Arabidopsis* PRs, mature LRs and emerged LRs shown in B and C are identical to those used in Fig. 1B.

The catenary parameter *a* is the reciprocal of the curvature of the dome controlling its sharpness [Fig. 3A, left panels; *y*=*a* cosh(*x/a*) – *a*], and works as a factor of the isometric scaling (i.e. geometrical similarity); by scaling both *x*- and *y*-coordinates with catenary parameter *a*, catenary curves commonly converge to the parameter-free catenary function [*Y=*cosh(*X*) − 1, *X=x/a*, *Y=y/a*; Fig. 3A, right panels]. The catenary parameter *a* also works as the isometric scaling factor to the catenary-closest ellipse, as each fitted value of ellipse parameters [*a_ellipse_* and *b_ellipse_*; *y*=*b_ellipse_*(1−(*x*/*a_ellipse_*)^2^)^0.5^] were both proportional to that of catenary parameter *a* among *Arabidopsis* PR and LR samples (Fig. S4K,L). The isometric scalability of catenary and catenary-closest ellipses predicts the isometric scalability of root tip outlines. that of the root tip outlines. Strikingly, the isometric scaling of each sample using its own fitted catenary parameter *a* (Fig. 3B) successfully normalized differences of the individual size among PR and LR samples (Fig. 3C, left panel), so that all root outlines commonly converged to the parameter-free catenary function (Fig. 3C, right panel). These results verified the isometrically scalable nature of *Arabidopsis* PR and LR.

To further examine the isometric scaling of dome-shaped outlines across diverse species, we analyzed the PRs of eight additional eudicot species and one monocot species (Fig. 3B,D, left panel; Fig. S5A). Regardless of their morphological diversity (i.e. size and aspect ratio of the dome, the number of ground-tissue layers, and the structure around the quiescent center, such as the open or closed meristem; Fig. S5B,C) (Clowes, 2000; Heimsch and Seago, 2008), the root tip outlines of all these species fitted to the catenary curve and the catenary-closest ellipse to a similar extent (averaged MSE in Fig. S6). The fitted value of catenary parameter *a* reflected the species-specific dome size (Fig. 3B; Fig. S4K,L). Moreover, the rescaled PR outlines by the fitted catenary parameter *a* converged universally to the parameter-free catenary curve (Fig. 3D, right panel) as in *Arabidopsis* PRs and LRs. These results indicate that the dome outline diversity of various angiosperm PRs, as well as *Arabidopsis* LRs, universally emerge from the isometric scaling by the species- and root class-specific catenary parameter *a*.

### Tissue growth rules underlie self-organized formation of the catenary-curved geometry in *Arabidopsis* LRs

**Fig. 4.**
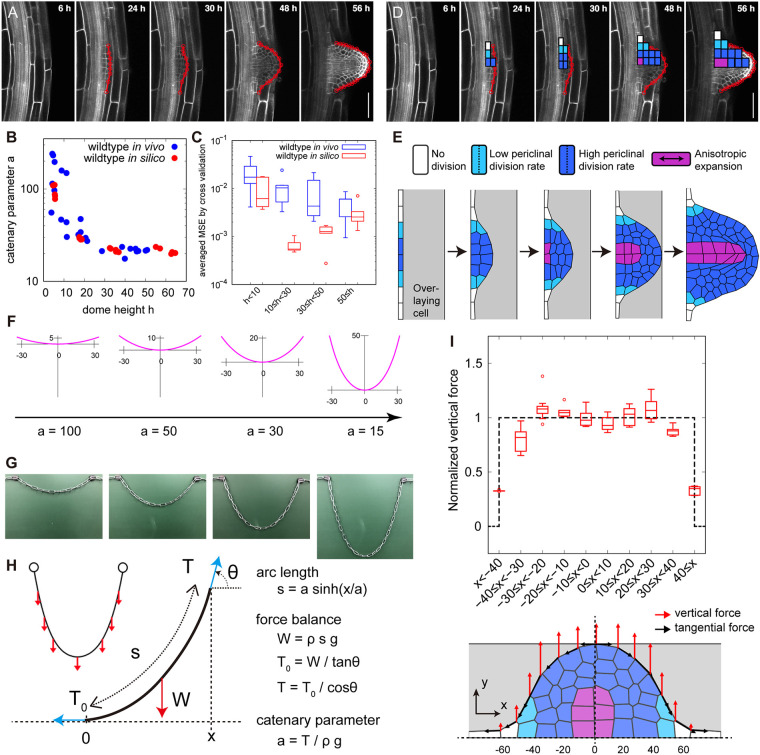
**Geometry and mechanics of a catenary-curved dome during LRP tissue growth.** (A) Longitudinal confocal sections from time-lapse imaging of a developing *Arabidopsis* LRP visualized using *35S:Lti6b-GFP* (a plasma membrane marker). The elapsed time (h) after gravistimulation for inducing LRP development is indicated in each panel. Red lines indicate LRP dome outlines. (B) A graph of catenary parameter *a* (*y*-axis) plotted against dome height *h* (*x*-axis) of growing LRP outlines quantified by the NLS method. (C) Cross validation test (Eqn 12) of *in vivo* and *in silico* LRP outlines fitted with catenary function. Averaged MSE (*y*-axis) against the dome height *h* (*x*-axis) *in vivo* [blue, *n*=10 (h<10), *n*=10 (10≤h<30), *n*=10 (30≤h<50), *n*=10 (50≤h)] and *in silico* (red, *n*=5 for each dome height range, h<10, 10≤h<30 and 30≤h<50) are shown. (D) Rules of cell divisions (white, no division; light blue, single division; deep blue, three consecutive divisions) and anisotropic cell expansion in the proximodistal direction (magenta, presence; other colors, absence) observed in the LRP development *in vivo*. (E) Tissue-mechanical simulation from a flat primordium to dome formation during LRP development with a mass of overlaying cells (gray). Cell division and expansion rules (color-coded as in D) were incorporated into the simulation. See also Movie 1. (F) Catenary curves of different parameter values *a* with its width (*x*-axis) approximately equal to that of an actual LRP (Fig. 4A). (G) Catenary curves formed by chains of increasing length with fixed ends under gravity. (H) The mechanics of the catenary curve; the gravity works as vertically uniform force *W* on the chain, and is balanced with the tangential tension *T* at the mechanical equilibrium. *s*, *a*, *x*, *ρ*, *g* and *θ* denote chain outline length, catenary parameter, *x*-coordinate of the catenary chain, mass density, the gravitational acceleration and the angle from horizontal *x*-axis, respectively. *ρg* represents the gravitational (vertical) force per unit length. (I) Distribution of vertical force (red arrows) and tangential force (black arrows) on dome outlines after cell expansion in five representative simulations (bottom panel shows a representative outcome; dashed black lines indicate the *x*- and *y*-axes). The magnitude of vertical force normalized by its spatial average over the dividing zone (dark blue and light blue cells in the bottom panel) plotted as a function of *x*-coordinate along the dome width (upper panel). Data are mean±s.d. of five independent simulations. The upper and lower hinges, and the middle lines of box plots in C and I represent the 25th, 75th and 50th percentiles, respectively. Scale bars: 50 µm.

The dome shape of LRs emerges from a developmental process (Goh et al., 2016; Lucas et al., 2013; Vermeer et al., 2014; von Wangenheim et al., 2016). Both young emerged LRs and mature LRs had almost identical values of the catenary parameter *a* (Fig. 3B), suggesting that the formation of the isometrically scalable dome shape is completed before the LR emergence. We further found that the dome outlines fit well to a catenary curve consistently from early (LRP dome height 10≤h<30, stage II-V), late (30≤h<50, stage VI-VII) and emerged LRP (dome height 50≤h, emerged, Fig. 4A-C), judged by the same level of averaged MSE as those of emerged LRs (Fig. 4C; Fig. S3). The value of catenary parameter *a* of LRP decreased with developmental progression, and eventually reached those of emerged LRs (Fig. 4B).

In order to gain insights as to what developmental processes contribute to the formation of the isometrically scalable dome and its maintenance, it is useful to refer to a developmental model of the catenary curve, i.e. a free-hanging chain stably forming with its own weight when its ends are supported (Fig. 2B, left panel) (Block et al., 2006; Lockwood, 1961); though, to our knowledge, a model for the catenary-closest ellipse has not been described so far. To this end, we performed tissue growth simulations of LRP development by focusing on the catenary-curved geometry.

We used the vertex model for mechanical deformation of cells (Materials and Methods; Farhadifar et al., 2007; Hamant et al., 2008; Honda, 1983; Uyttewaal et al., 2012) by incorporating tissue growth rules of LRP (i.e. the rate and orientation of cell division and expansion) obtained from the previously reported time-lapse imaging of wild-type LRP development (Fig. 4A,D,E; Materials and Methods; Goh et al., 2016; von Wangenheim et al., 2016). Briefly, in the early phase in which a four-layered primordium (stage I to IV) is formed, one anticlinal and three periclinal divisions occurred synchronously in the central region of a primordium (dark blue cells, Fig. 4D,E), and one periclinal division occurred in the flanking region (light blue cells, Fig. 4D,E), whereas no division occurred at the periphery of the primordium (white cells, Fig. 4D,E; Movie 1). In later phases, anisotropic cell expansion occurred locally at the central bottom cells (pro-vascular cells shown in purple, Fig. 4D,E), and the LRP subsequently emerged through the overlaying cells (Goh et al., 2016; von Wangenheim et al., 2016). Importantly, simulations incorporating the tissue growth rules quantitatively reproduced the catenary-curved geometry of a growing LRP dome (Fig. 4E). Even in the absence of overlaying cells at the earlier stages, the catenary-curved dome develops in simulations (Fig. S7A), though the shape reproducibility was less pronounced than those produced in the simulations with overlaying cells (Fig. S7A-C). These results recapitulate the decrease of the catenary parameter *a* along the course of LRP development (Fig. 4B), with similar or even a higher degree of fitness compared with those observed *in vivo* (Fig. 4C).

### Tissue growth rules of LRP account for the mechanics of catenary curve formation in *Arabidopsis*

Catenary-curved hanging chains and bridges (Fig. 2B) are load-bearing structures that follow the mechanical equilibrium between gravity (i.e. vertical and uniform force distribution) and tangential tension on the chain (Fig. 4F-H; Block et al., 2006; Lockwood, 1961). Geometrical similarity between catenary chains and LRP domes prompted us to examine whether tissue growth behaviors in LRP account for the mechanics of their catenary curves. To this end, we decomposed the force along the dome outline into the vertical and the tangential components (red and black arrows, respectively, in Fig. 4I, lower panel) at the mechanical equilibrium during the tissue growth simulations. The vertical force was uniform at the central domain but sharply decreased to zero in the peripheral region of the primordium (Fig. 4I, upper panel). The tangential force was the lowest at the dome center and increased toward the peripheries with inverse proportionality to the cosine of the tangential angle (Fig. S7D). The spatial distribution of vertical and tangential forces on the LRP outlines was consistent with that of the gravity and tangential tension of catenary chains, respectively (Fig. 4I; Fig. S7D). Thus, our simulations also support tissue growth behavior of LRP for the mechanics of catenary curve formation.

The mechanical and geometrical features of growing LRP (Fig. 4A-E,I) agreed with those assumed for a hypothetical catenary chain of extending length (Fig. 4F-H), in which (1) both ends are fixed, resulting in the sharp boundary of force distribution while (2) its outline length increases under gravity. This consistency suggests that (1) the sharp boundary and (2) unidirectional and uniform force distribution are necessary for the formation of a catenary-curved LRP dome. The two elementary candidate rules of tissue growth are that there is (1) an occurrence of periclinal divisions of the cells at the central domain of LRP and a lack of cell division at the peripheral edge of LRP, and that there is (2) a spatially uniform occurrence of unidirectional (i.e. anisotropic) tissue growth via periclinal divisions and/or cell expansions at the central domain (Fig. 4D,E).

### Sharp boundary of periclinal cell division rate was required for catenary curve formation

**Fig. 5.**
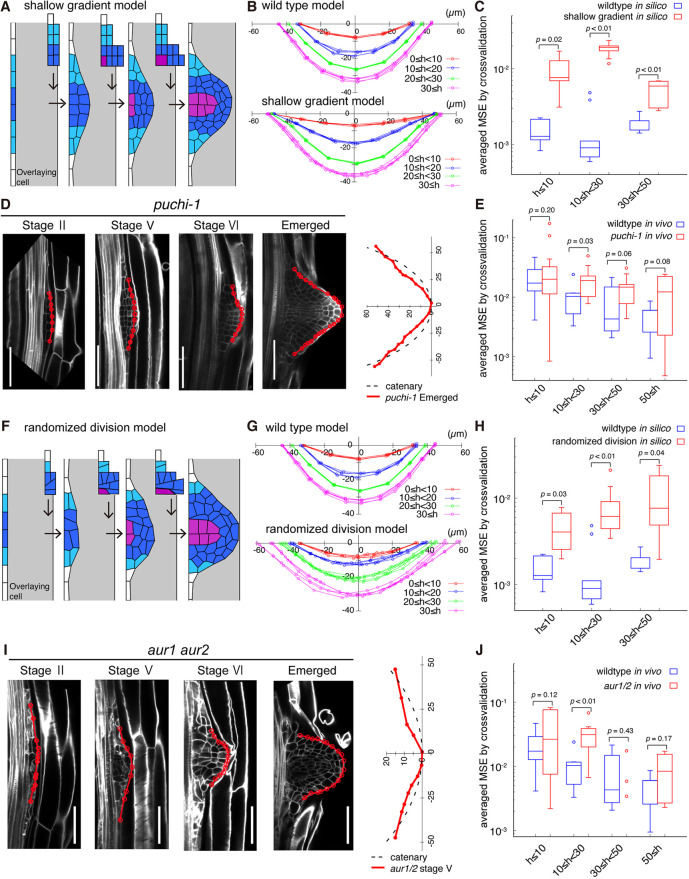
**Localized periclinal cell divisions of LRP determine its dome shape.** (A,F) Simulation of (A) the shallow gradient model assuming supernumerary cells in the flanking region (light blue), and (F) the randomized division model assuming randomized cell division orientation in the central domain (dark blue). Division and expansion rules of remaining cells were left unchanged from those used in Fig. 4E. Panels from left to right correspond to LRP shapes observed *in vivo* at h<10, 10≤h<20, 20≤h<30 and 30≤h<50. See also Movie 1. (B,G) Dome outlines during the *in silico* simulation of the shallow gradient model (B) and the randomized division model (F). The dome outlines of wild-type templates are derived from Fig. 4E. Colors denote root dome height (µm). (C,H) Averaged MSE from the cross-validation test (Eqn 12) with the catenary curve in simulations (*in silico*) of wild-type template (blue circles), the shallow gradient model (red circles in C; *n*=5 for each dome height range, h≤10, 10≤h<30 and 30≤h<50) and the randomized division model (red in H; *n*=5 for each dome height range, h≤10, 10≤h<30 and 30≤h<50). (D,I) Longitudinal confocal sections of LRP at different developmental stages in *puchi-1* (D) and *aur1 aur2* (I) mutants (left panels), and their dome outlines plotted in the cartesian coordinate together with an imaginary fitted catenary curve (dotted black line, right panels). Cell walls were stained with SR2200 (white). Red lines and circles indicate LRP dome outlines and cell junctions, respectively. Scale bars: 50 µm. (E,J) Averaged MSE from the cross-validation test with catenary curves for *Arabidopsis* LRP of wild type (blue circles), *puchi-1* [red circles in E; *n*=21 (h≤10), *n*=9 (10≤h<30), *n*=9 (30≤h<50), *n*=7 (50<h)] and *aur1 aur2* mutants [red circles in J; *n*=9 (h≤10), *n*=10 (10≤h<30), *n*=3 (30≤h<50), *n*=4 (50≤h)]*.* The upper and lower hinges, the middle lines and error bars of box plots in C, E, H and J represent the 25th, 75th and 50th percentiles, and s.d., respectively. Date sets for wild type used in C, E, H, J were identical to those in Fig. 4C. Statistical significance was determined using Welch's unpaired, one-tailed *t*-test.

To examine whether (1) the periclinal divisions of the cells at the central domain and the lack of cell division at the peripheral edge is indispensable for catenary curve formation, we first perturbed distribution of cell division rates within an LRP in simulations. In addition to the naturally occurring situation in which a single cell layer with a low periclinal cell division rate constitutes a sharp boundary separating a rapidly dividing central region from a mitotically quiescent outer region (Fig. 4D,E), we also simulated a hypothetical situation in which multiple cell files with a low periclinal cell division rate were assumed, between the central and peripheral regions (light blue cells in Fig. 5A), to make a shallow division rate gradient (Fig. 5A; Movie 1; shallow gradient model). In the latter case, the simulated dome outline became more extended towards the periphery than wild type (30<h at |*x*|>40 in Fig. 5B), and deviated from the catenary curve even after incorporating the anisotropic cell expansion (Fig. 5C). This deviation became further exaggerated when it was assumed that there were more cell files with a low division rate (shallower gradient model, Fig. S8A,B,D). Additionally, the spatial distribution of vertical force was less uniform and continuously decreased from the central to the peripheral domain (Fig. S8F,G, left panel). On the other hand, increasing the number of cell files with a high division rate at the central domain (dark blue cells) to make a sharper boundary resulted in an outline that can be robustly fitted to a catenary curve with a larger value of the catenary parameter (increasing central zone model, Fig. S8C,E). These simulations predicted that the sharp boundary of cell division rate at the flanking region is required for catenary curve formation.

We experimentally verified the requirement of the sharp boundary of cell division rate by using the *Arabidopsis puchi-1* mutant defective in a gene encoding the auxin-inducible AP2/EREBP-type transcription factor PUCHI (Hirota et al., 2007) (Fig. 5D). The *puchi-1* mutant lost the sharp boundary due to the extra periclinal divisions at the flanking region significantly increasing the number of cell files with more than one cell layer compared with those in wild type, whereas the cell files with more than two cell layers did not increase (Fig. S9A,B) (Hirota et al., 2007). This defect specifically increased the number of cell layers at the flanking region, substantiating the shallow gradient of periclinal division rate assumed *in silico* (Fig. 5A). Intriguingly, the emerged LRP dome of the *puchi-1* mutant appeared to be more tail-extended (Fig. 5D), and thereby deviated from a catenary curve (i.e. averaged MSE higher than that of wild type in Fig. 5E). The consistency between the dome outlines of the *puchi-1* mutant *in vivo* and the simulations of shallow gradient *in silico* confirmed that the sharp boundary of division activity at the flanking region of LRP is required for catenary curve formation.

### Anisotropic and uniformly-distributed tissue growth contributes to catenary curve formation

To examine whether (2) the spatially uniform occurrence of periclinal division is indispensable for the formation of the catenary-curved dome shape, we randomized the cell division orientation in simulations (Fig. 5F; Movie 1, randomized division model). The dome outline became less symmetric in the bilateral axis, as seen for the displacement of the dome tip from the center, and thereby deviated from a catenary curve (h<30 in Fig. 5F-H). The spatial distribution of vertical forces was accordingly less uniform (Fig. S8F,G, right panel).

We experimentally verified the requirement of the anisotropic tissue growth arising from the periclinal cell division by using the *Arabidopsis aur1 aur2* mutant, in which the two AURORA kinase genes indispensable for the correct positioning of the cell division plane in the LRP were disrupted simultaneously (Van Damme et al., 2011). In the *aur1 aur2* mutant, division-plane orientation (angle), especially at the foot of the LRP, was significantly more variable than in wild type (at the central domain of stage V in Fig. 5I and Fig. S9A,C) (Van Damme et al., 2011; von Wangenheim et al., 2016), substantiating the simulation with a randomized division orientation described above (Fig. 5F). In the early stages (stage II and V in Fig. 5I), the *aur1 aur2* mutant consistently lost bilateral symmetry in their LRP outline, resulting in the deviation from a catenary curve (significantly higher MSE than in wild type; h<30 in Fig. 5J). The consistent defects in the dome outlines between the *aur1 aur2* mutant and the simulations with randomized cell division orientation confirmed that anisotropic and uniformly distributed tissue growth arising from the periclinal division was required for the catenary curve formation. Importantly, the fitness of *aur1 aur2* mutant root tip outline to a catenary curve improved as LRP developed and became similar to that of wild type (30≤h in Fig. 5I,J). This observation further supports the hypothesis that the anisotropic cell expansion at the central domain, which occurs in both wild-type and *aur1 aur2* LRP (Fig. 4D,E; Fig. 5F,I), promotes (2) the anisotropic tissue growth and hence the catenary curve formation.

## DISCUSSION

### Isometric scaling of plant root tip morphologies into a universal catenary curve

It has long been acknowledged that organ morphologies are conservative while being diverse among species, depending on survival strategies and adaptation to the environment. Despite the morphological diversity of root tip in size (width) and internal cellular organization (Fig. S5) (Clowes, 2000; Hamamoto et al., 2006; Heimsch and Seago, 2008), our morphometric analysis revealed that the outlines of the PRs of ten angiosperm species and *Arabidopsis* LRs commonly fitted to a catenary curve and its essentially equivalent curve, a catenary-closest ellipse (Fig. 2A; Figs S3,S4,S6). The catenary curve is seen in free-hanging chains and bridges (Fig. 2B), and has several interesting features in mathematics, physics and architecture, such as (1) being represented by a simple mathematical function with a single catenary parameter [*y*=*a* cosh(*x/a*) − *a*)], (2) being stably formed under gravity in a free-hanging chain; and (3) being widely used in various architectures for its structural stability.

Each outline shape of root tips across root class and species is surprisingly represented by a single catenary parameter *a* (Fig. 3B, lower panels), which is the reciprocal of the dome curvature (Fig. 3B, upper panels), and also the tangential tension divided by the vertical force per unit length (Fig. 4H), representing both geometry and mechanics. From a mathematical interpretation, the catenary parameter works as a factor of an isometric scalability; i.e. each catenary curve is able to superimpose on a universal parameter-free catenary curve via transforming equally on *x*- and *y*-coordinates with the catenary parameter (Fig. 3A). Indeed, by the isometric scaling, all root tip outlines superimposed to the parameter-free catenary function (Fig. 3C,D). Other known examples of conservative organ outlines [e.g. the human skull; the cannon bone of ox, sheep and giraffe (Thompson, 1917); the beaks of songbirds (Abzhanov, 2017; Campas et al., 2010)] superimpose among neighboring species via affine transformations, which allow the transformation of the outlines on *x*- and *y*-coordinates with different rates and/or different directions of deformation (Campas et al., 2010; Fritz et al., 2014; Thompson, 1917). This indicated that the isometric scalability of plant root tip outlines is a previously undescribed highly constrained solution for the conservative morphologies, and suggested underlying constraints during development.

The isometric scalability also mathematically ensures the reproducibility of the rescaled outline shape (Fig. 3A). This is distinct from a recently reported mechanism of shape reproducibility within species via spatiotemporal averaging of variable cell growth during organogenesis (Hong et al., 2016). Despite the distinct mechanisms for the reproducibility, the shape of *Arabidopsis* PR and LR tips were highly reproducible (1-3% in Fig. 1D,E) at a level similar to *Arabidopsis* sepals (≃5%) (Hong et al., 2016). To date, morphological diversity among species (Abzhanov, 2017; Thompson, 1917) and shape reproducibility within a given species have been studied rather independently (Hervieux et al., 2017; Hong et al., 2016, 2018). The isometric scalability adequately achieves both diversity and reproducibility, recapitulating the conservative feature of organ morphologies. The general methodology established in this study (Fig. 3) provides a way to unravel the isometric scalability in other biological shapes.

### Developmental constraints for the formation and maintenance of a catenary-curved dome

The geometry and mechanics of growing LRP (Fig. 4A-E,I) are consistent with those of a hypothetical catenary chain of extending length (Fig. 4F-H), which stably forms under (1) the sharp boundary and (2) unidirectional and uniform force distribution, such as gravity. These mechanical consistencies proposed the following developmental constraints for the formation of a catenary-curved dome: (1) unidirectional (i.e. anisotropic) tissue growth localized at the central domain of LRP, with a lack of growth at the peripheral edge of LRP; and (2) the spatially uniform occurrence of anisotropic tissue growth via periclinal divisions and/or cell expansions at the central domain (Fig. 4D,E). These two tissue growth rules successfully recapitulated the spatial distribution of the force field that is predicted for the catenary-curved chain (Fig. 4G-I). The first constraint, (1) the localized occurrence of the anisotropic tissue growth, was verified using the *Arabidopsis* *puchi-1* mutant, which lost the sharp boundary due to the extra periclinal divisions at the flanking region (Fig. 5D; Fig. S9A,B), resulting in a tail-extended dome shape deviated from a catenary curve (Fig. 5D,E). The cell divisions in the peripheral regions of LRP are strongly repressed by the locally expressed genes represented by *PUCHI*, and this restriction plays critical roles to define the organ boundaries and organ outgrowth (Hirota et al., 2007; Lavenus et al., 2015; Torres-Martinez et al., 2019; Trinh et al., 2019). Our findings further demonstrated the importance of the peripheral region for the outline morphology of LRP. The second constraint, (2) spatially uniform occurrence of anisotropic tissue growth, was verified using an *Arabidopsis aur1 aur2* mutant, in which the division-plane orientation was varied (Fig. 5I; Fig. S9A,C). A less symmetric dome shape deviated from a catenary curve during the early LRP stages indicated the necessity of the uniform occurrence of periclinal divisions. On the other hand, developmental convergence of the LRP outlines to the catenary curve from later stage onwards also supports the significance of uniform occurrence of the anisotropic cell expansion, which was normal in this mutant (Fig. 5I). These constraints are also consistent with the notion obtained in previous studies; a small set of cell division rules reflecting cell geometry promotes periclinal divisions in the growing LRP (von Wangenheim et al., 2016). Additionally, upon LRP emergence, mechanical constraint from the overlaying tissues affects the LRP shape and its reproducibility, perhaps through controlling the potential growth pattern (Fig. 4E; Fig. S7A-C) (Lucas et al., 2013; Vermeer et al., 2014). Taken together, we propose that the spatiotemporal regulation of tissue growth at the central or peripheral region, under the control of specific sets of regulators (Lavenus et al., 2015; Torres-Martinez et al., 2019), is the developmental constraint for the catenary-shaped root tip in LRP development.

The catenary parameter is stabilized around the emergence stage of LRP development, in which the RAM is established in preparation for successive cell proliferation to extend LRs (Fig. 4A-C; Fig. S1B,C; Goh et al., 2016; von Wangenheim et al., 2016), and further maintained in the matured LRs (Fig. 3B). Interestingly, previously reported growth simulations based on LR and PR cell geometries predicted that a localized and uniform occurrence of anisotropic tissue growth at the RAM was required for maintaining organ outline morphology, as well as the cellular organization (Hejnowicz, 1984; Nakielski and Lipowczan, 2013; Szymanowska-Pułka et al., 2012), and the cellular geometry within the root tip of the embryo is the mechanical constraint on tissue growth (Bassel et al., 2014). We hypothesized that the tissue growth rules of RAM might fulfill the developmental constraint for catenary-shaped dome formation through anisotropic growth via oriented cell divisions and expansion, and for maintaining a largely constant width. Furthermore, the structural feature of RAM is essentially conserved across vascular plants under the control of shared regulatory mechanisms (Huang and Schiefelbein, 2015). Therefore, it will be interesting to study in future whether (1) the localized and (2) spatially uniform occurrence of anisotropic tissue growth are shared constraints for the maintenance of a catenary curve across the root classes and species. Underpinning of the constraints by the cell wall extensibility and the turgor pressure of individual cells may be also predicted in future, if the formulation proposed by Lockhart (1965) and the elasto-plastic cell deformation (Geitmann and Ortega, 2009; Ortega, 1985) are to be incorporated into the present vertex model.

The catenary curve becomes a three-dimensional dome surface when it is rotated (around the *y*-axis), as seen in the root tip with a rotational symmetry (Fig. S1A), and such three-dimensional shapes are also used in architectures of various sizes and materials [e.g. St Paul's Cathedral (Heyman, 1998) and snow igloos (Handy, 1973)]. Three-dimensional root tip shapes have been previously shown to affect the penetration ability of roots into soil in wheat (Colombi et al., 2017), or into a hard medium in *Arabidopsis* (Roue et al., 2020). In addition, an engineering approach using soft robots suggested that plant root tip morphology governs the penetration stress and the efficient elongation in soil (Mishra et al., 2018). Our simulations indicated that the mechanical force produced by the tissue growth was uniformly distributed on the surface of the catenary-curved root tips (Fig. 4G-I; Fig. S8F,G). This finding encourages us, in future, to test whether the force produced by the interaction between the root tip and soil is also uniformly distributed onto the entire tip surface, and thereby contributes to the efficient penetration of roots into soil.

## MATERIALS AND METHODS

### Plant materials and growth conditions

We used the Col-0 wild type accession for analysis of root tip shape in *Arabidopsis*. For analysis of multiple species, we selected one monocot (*Allium fistulosum*, Welsh onion), three rosids (*Cucumis sativus*, cucumber; *Viola mandshurica*, violet; *Arabidopsis*), one caryophyllales (*Dianthus superbus*, pink) and five asterids (*Primula polyantha*, primrose; *Cosmos bipinnatus*, common cosmos; *Daucus carota*, carrot; *Antirrhinum majus*, snapdragon; *Nemophila menziesii*, nemophila). All seeds except for *Arabidopsis* were obtained commercially (Sakata Seed Corporation). *puchi-1* (Hirota et al., 2007), *aur1*-*2* (SALK_031697) and *aur2-2* (GK403B02) (Van Damme et al., 2011) have been described previously. The *35S::LTI6b-GFP* line (CS84762) was obtained from the *Arabidopsis* Biological Resource Center. Seeds were surface sterilized and sown on 1× MS medium solidified with 0.4% gellan gum, containing 1% (w/v) sucrose, or on *Arabidopsis* growth medium (Okada and Shimura, 1992) supplemented with 1% (w/v) sucrose and 1% (w/v) agar.

### Imaging and image processing

For LR analysis, 7-day-old seedlings were fixed with 4% (w/v) paraformaldehyde for 30 min at room temperature, washed twice with PBS and then cleared with ClearSee solution, including 0.2% (v/v) SCRI Renaissance 2200 (SR2200) for cell wall staining (Kurihara et al., 2015; Musielak et al., 2015). Images were obtained using a Nikon C2 confocal microscope with a 405-nm laser line for excitation of SR2200. For PR analysis, seedlings were grown for several days until the first LR appeared and were then observed using a confocal microscope after staining with propidium iodide. Images were obtained using a Zeiss LSM710 confocal microscope. Image segmentation was performed to detect the shape and position of each cell using the Fiji plugin Tissue Analyzer (Aigouy et al., 2010).

For time-lapse observation of LRP development, 4-day-old seedlings (*35S::LTI6b-GFP*) grown vertically were transferred into a coverglass-bottomed chamber (LabTek, Thermo Fisher Scientific) with a block of solid medium. Images of an identical primordium were obtained 6, 24, 30, 48 and 54 h after gravistimulation with a Nikon C2 confocal microscope, and processed with ImageJ software.

### Determination of root tip outlines and unification of the coordinate system

We analyzed the vertical sections of the angiosperm PR tips (Fig. 1A; Fig. S1A-C, Fig. S5A) and *Arabidopsis* LR tips at different developmental stages (Fig. 1A). For both the PR and LR tips, we determined the outline from the positions of the cell junctions along the dome surface (Fig. S1D,E). For the PRs, we analyzed the region from root tip to the boundary between the meristem and elongation zone, except for the sloughing root cap layer (red dotted box in Fig. S1D). The *x*- and *y*-axes were set parallel to the upper boundary at the opposite side of the tip and the proximal-distal axis of the dome, respectively. In order to compare different root tip outlines, it is necessary to objectively unify the coordinate system with setting the peak of the tip at origin. Therefore, we defined the origin of the coordinate system by the following three steps (see also Fig. S1F): (1) the junction points on the outline were duplicated and turned 180° (π radian); (2) the duplicated positions were translated to satisfy that the maximum *y* of the original position was equal to the minimum *y* of the duplicated position; and (3) the origin of *x*- and *y*-coordinates were determined as the mean of *x* of all points and the minimal *y* of the original points, respectively. Given this unified coordinate system, the dome area and width at a height from the root tip are comparable (Fig. S1G).

### Radial Fourier series expansion

The radial Fourier series expansion enables the characterization of the shape of a closed curve using the radial information of the polar coordinate system (*r*_*i*_, *θ*_*i*_) derived from the cartesian coordinate system (*x_i_*, *y_i_*) (Hong et al., 2016). The closed curve of root tip outlines was prepared by the methods described above, (1) duplication and (2) translation, without loss of generality. In this set up, *i* denotes the label of all junction points, including the duplicated ones (Fig. S1F) up to the total number of duplicated junction points (*i*=1, 2, · · · , *M*; 0≤*θ*_*i*_<2*π*). The radial Fourier series expansion *r*(*α*) can be decomposed as,(1)
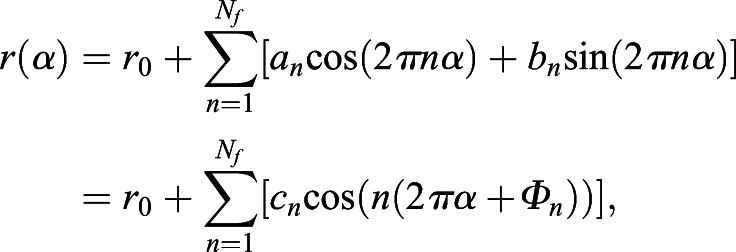
(2)
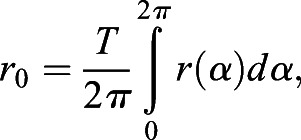
(3)
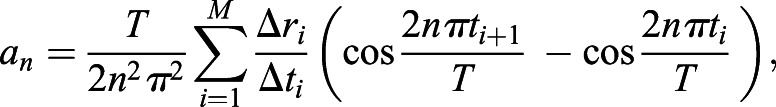
(4)
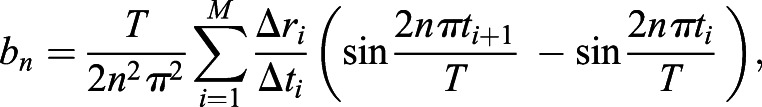
where *α*(0≤*α*≤1) denotes a continuous normalized perimeter along the outline. *Φ*_*n*_ and *c*_n_ denote the angular phase and the amplitude, respectively, of the *n*-th Fourier mode. Δ*t*_*i*_ denotes the normalized perimeter at the point (*r*_*i*_, *θ*_*i*_). Δ*r*_*i*_ denotes the radial distance between two successive junction points, and *N_f_*=200 in this study. In the explicit form, *α* is equal to *t*_*i*_/T with the total perimeter of the outline *T*, and Δ*t*_*i*_ and Δ*r*_*i*_ can be defined as,(5)
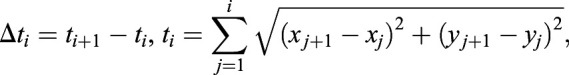
(6)
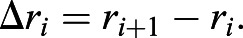


The shape is characterized by normalized radial Fourier series expansion,(7)
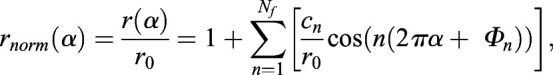
to eliminate the influence of the size on the shape. For the normalized radial Fourier expansion 
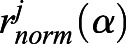
 of the sample *j* (*j*=1, 2, · · · , *K*), where *K* stands for the total number of samples, the sample average with continuous outline can be calculated as(8)
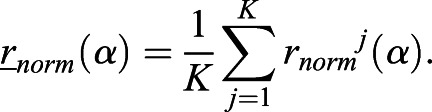


An indicator of shape reproducibility *S_2_^1/2^*, representing normalized error of shape, can be evaluated by the root mean squared deviation from the sample-averaged normalized shape(9)
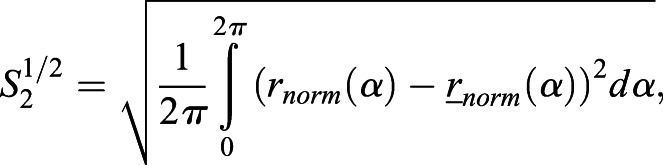
where 
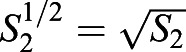
, as described previously with regards to sepal shape (Hervieux et al., 2017; Hong et al., 2016).

### Statistical analysis of the dome shape

We fitted the outline of the dome to the following five functions: parabola (*y* = *a*_1_*x*^2^), catenary (*y* = *a*_2_ cosh(*x*/*a*_2_)−*a*_2_), ellipse 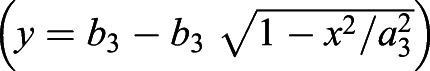, hyperbola 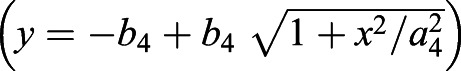, and cosine (=−*a*_5_ cos(*b*_5_*x*) + *a*_5_), where *a_i_* (*i=*1, 2, 3, 4, 5) and *b_i_* (*i=*3, 4, 5) are fitting parameters. The hyperbola has a common mathematical nature (i.e. conic section) to parabola and ellipse; catenary and cosine are mechanically stable functions under unidirectional force (Block et al., 2006; Lockwood, 1961) (Fig. 2B) and the Euler buckling (Timoshenko and Gere, 1961), respectively. Using these functions, we applied the non-linear least-squares (NLS) method (Moré, 1978) to each dataset of normalized cell junction positions on the outlines of multiple LRP samples at each developmental stage, and PR samples from each species. We evaluated the positional variation of the dome outline among samples on the basis of the SSE of the *y*-coordinate (height) from these functions defined by(10)
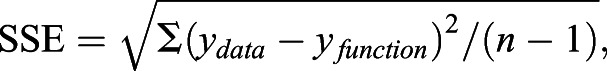
and the AIC defined by(11)
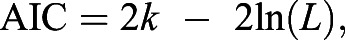
which is the number of parameters in the model *k* (one for parabola and catenary, and two for the other three functions) minus the natural logarithm of the maximum likelihood *L* (Akaike, 1974; Burnham et al., 2002; Sakamoto et al., 1986). The AIC is one of the most popular and statistically rigorous criteria, as the AIC of the best-fit function takes the minimum value. We computed ΔAIC defined as the difference in AIC between a given model function and the lowest AIC model function. Thus, the fitting function for which ΔAIC=0.0 is the best model, whereas models with larger ΔAIC values are not as good. Generally, models with ΔAIC<2.0 have the potential to be the best model, and those with ΔAIC<7.0 cannot be easily rejected (Burnham et al., 2002). We performed NLS-fitting and AIC calculation with the R interface using the minpack.lm package (Elzhov et al., 2015). We also performed the cross-validation test, which is one of the model validation techniques, providing us with a measure of how robustly the model will predict the data set without overfitting or selection bias (Hong et al., 2016). In the test, a portion of the data points (referred to as the testing set) is validated based on the rest of the data points (referred to as the training set). In our case, the training set was 99% of the junction points that were randomly selected, whereas the testing set was the remaining 1%. The model validation was evaluated by the MSE between the fitting function from the training set and the testing set. Applying this process to different training set *i* with different random seeds for *N* times, the final validation is performed by(12)
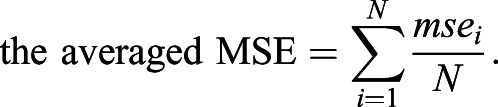


### Formulation of tissue-mechanical simulations

The cell vertex model is useful for simulating the mechanical deformation of cells in tissues based on the forces acting on each cell, in which the cell configurations are described as polygons that have vertices that form cell junctions subjected to mechanical force (Farhadifar et al., 2007; Honda, 1983). Cells change their shape based on the force balance represented as mechanical energy *E* with dimensionless time and mass. The model is represented here by the ordinary differential equations of the position vector 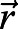 of each vertex:(13)
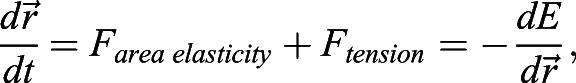
(14)
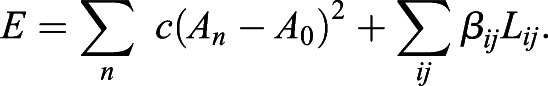


The area elasticity *F_area elasticity_* is exerted on a vertex *i* by the cell face *n* to which the vertex *i* belongs, while the cell area *A_n_* approaches the target area of *A*_0_ with normalized strength of the elasticity. The tension *F_tension_* is exerted on a vertex *i* by the connecting edges between vertices *i* and *j*, where *F_tension_* increases as the edge length between vertices *i* and *j* (*L_ij_*) increases, depending on the cell-wall extensibility *β_ij_*. For all cells, we set *c*=1 μm^−2^, *β_ij_*=0.002 µm for horizontal edges, and *β_ij_=*0.004 µm for vertical edges, which reproduced the average cell area and cell aspect ratio of *Arabidopsis* wild-type cells in LRP at developmental stage IV (Goh et al., 2016). In addition, plant cells undergo plastic deformation by the irreversible cell growth, which has been formulated by multiplication of the excess turgor pressure over yield stress and the cell wall extensibility of cell edge (Lockhart, 1965). In the present model, the plastic deformation was formulated by the irreversible increase of the target area of cells, as described in detail below, instead of the edge length in the Lockhart model (Lockhart, 1965). We integrated the cell vertex model numerically using the Euler method and confirmed that the obtained results were not greatly influenced by the choice of the temporal discretization size *dt*.

### Cell division and expansion in simulations

For the initial condition of the vertex model, 20 cells were arranged horizontally (only 8 to 12 cells at the central region shown in Figs 4,5, Figs S7, S8 and Movie 1); the four cells (dark and light blue in Fig. 4E) among the eight at the center subsequently divided, but the others did not divide (white in Fig. 4E) during the wild-type simulation. For the boundary conditions, the vertices at the basal end of the tissue could be displaced horizontally but not vertically (i.e. fixed at *y*=0) to mimic the high stiffness of the adjacent parental xylem cells, whereas those at the apical end were displaceable in any direction. Below 40 μm of the LRP height, those at the apical end are adjacent to an imaginal cell to mimic the overlaying parental cells with vertical thickness 36 µm (*A*_0_=36 µm×20 cells×14 µm/cell=10,080 μm^2^; gray in Figs 4,5, Fig. S8 and Movie 1). All the vertices at both ends in the horizontal direction were fixed (at *x*=−140 μm and *x*=140 μm, respectively).

During the stages with one to four layers, the timing and orientation (periclinal/anticlinal) of cell divisions were set following the typical division rules in wild-type LRP (Goh et al., 2016; von Wangenheim et al., 2016). First, two central cells (dark blue in Fig. 4E) simultaneously underwent periclinal division. Second, these four central cells underwent anticlinal division, while two flanking cells (light blue cells, Fig. 4E) immediately outside those four cells simultaneously underwent periclinal division. Third, the eight central cells (dark blue in Fig. 4E) synchronously underwent two periclinal divisions, although cells at the outer layer divided a little earlier than cells at the inner layer of *Arabidopsis* LRP (Goh et al., 2016; von Wangenheim et al., 2016). The cell division plane was assumed to pass through the geometric center of the dividing cell with a small rotational variation in the periclinal/anticlinal direction, which followed the Gaussian distribution with an s.d. of 0.1 degree. Cells (dark and light blue in Fig. 4E) followed linear growth in wild-type LRP (Goh et al., 2016); immediately after the previous division event, the target area *A*_0_ was initially set as an average of 56 μm^2^ in cells, and temporally linearly increases with 
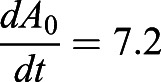
μm^2^/h. The cell division occurred when the cell area *A_n_* (Eqn 14) became twice that of the initial target area, with a variation following a Gaussian distribution with an s.d. of 5.6 μm^2^. New target area *A*_0_ was set to half of *A_n_* before the division event. After the four layers stage, cells (dark blue in Fig. 4E) were additionally divided until the cell numbers were identical to wild-type stage VII LRP (Goh et al., 2016; von Wangenheim et al., 2016), where cell expansion and division cycle were the same as the above, whereas the division plane was set to the short axis of the mother cell with a rotational variation following a Gaussian distribution with an s.d. of 0.1 degree.

To recapitulate the anisotropic growth of provascular bundle cells (Fig. 4A,D, right panel), we introduced rapid anisotropic expansion of the two central basal cells (magenta in Fig. 4E) by linearly increasing *A*_0_ with 
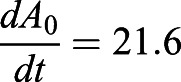
μm^2^/h and the same division cycle as above, and decreasing *β_ij_* of the vertical cell edge (*β_ij_=*0.002 μm at the end of four layers to *β_ij_=*0.0005 µm at the end of provascular cell expansion) over time, while keeping *β_ij_*=0.002 µm of the horizontal cell edge.

## Supplementary Material

Supplementary information

Reviewer comments

## References

[DEV196253C1] Abzhanov, A. (2017). The old and new faces of morphology: the legacy of D'Arcy Thompson's ‘theory of transformations’ and ‘laws of growth’. *Development* 144, 4284-4297. 10.1242/dev.137505 29183941

[DEV196253C2] Aigouy, B., Farhadifar, R., Staple, D. B., Sagner, A., Röper, J.-C., Jülicher, F. and Eaton, S. (2010). Cell flow reorients the axis of planar polarity in the wing epithelium of Drosophila. *Cell* 142, 773-786. 10.1016/j.cell.2010.07.042 20813263

[DEV196253C3] Akaike, H. (1974). A new look at the statistical model identification. *IEEE Trans Automat Contr.* 19, 716-723. 10.1109/TAC.1974.1100705

[DEV196253C4] Bassel, G. W., Stamm, P., Mosca, G., Barbier de Reuille, P., Gibbs, D. J., Winter, R., Janka, A., Holdsworth, M. J. and Smith, R. S. (2014). Mechanical constraints imposed by 3D cellular geometry and arrangement modulate growth patterns in the Arabidopsis embryo. *Proc. Natl. Acad. Sci. U.S.A.* 111, 8685-8690. 10.1073/pnas.1404616111 24912195 PMC4060677

[DEV196253C5] Block, P., DeJong, M. and Ochsendorf, J. (2006). As hangs the flexible line: equilibrium of masonry arches. *Nexus Netw J.* 8, 13-24. 10.1007/s00004-006-0015-9

[DEV196253C6] Burnham, K. P., Anderson, D. R. and Burnham, K. P. (2002). *Model Selection and Multimodel Inference: a Practical Information-Theoretic Approach*, 2nd edn. New York: Springer.

[DEV196253C7] Campas, O., Mallarino, R., Herrel, A., Abzhanov, A. and Brenner, M. P. (2010). Scaling and shear transformations capture beak shape variation in Darwin's finches. *Proc. Natl. Acad. Sci. U.S.A.* 107, 3356-3360. 10.1073/pnas.0911575107 20160106 PMC2840476

[DEV196253C8] Clowes, F. A. L. (2000). Pattern in root meristem development in angiosperms. *New Phytol.* 146, 83-94. 10.1046/j.1469-8137.2000.00614.x

[DEV196253C9] Colombi, T., Kirchgessner, N., Walter, A. and Keller, T. (2017). Root tip shape governs root elongation rate under increased soil strength. *Plant Physiol.* 174, 2289-2301. 10.1104/pp.17.00357 28600344 PMC5543947

[DEV196253C10] Darwin, C. (1859). *On the Origin of Species by Means of Natural Selection, or, The Preservation of Favoured Races in the Struggle for Life*. London: J. Murray. PMC5184128 30164232

[DEV196253C11] Di Ruocco, G., Di Mambro, R. and Dello Ioio, R. (2018). Building the differences: a case for the ground tissue patterning in plants. *Proc. Royal Soc. B* 285, 20181746. 10.1098/rspb.2018.1746 PMC6235038 30404875

[DEV196253C12] Elzhov, T. V., Mullen, K. M., Spiess, A.-N. and Bolker, B. (2015). minpack.lm: R Interface to the Levenberg-Marquardt Nonlinear Least-Squares Algorithm Found in MINPACK, Plus Support for Bounds.

[DEV196253C13] Eshel, A. and Beeckman, T. (2013). *Plant Roots: the Hidden Half*, 4th edn. Boca Raton, FL: CRC Press.

[DEV196253C14] Farhadifar, R., Röper, J.-C., Aigouy, B., Eaton, S. and Jülicher, F. (2007). The influence of cell mechanics, cell-cell interactions, and proliferation on Epithelial packing. *Curr. Biol.* 17, 2095-2104. 10.1016/j.cub.2007.11.049 18082406

[DEV196253C15] Fritz, J. A., Brancale, J., Tokita, M., Burns, K. J., Hawkins, M. B., Abzhanov, A. and Brenner, M. P. (2014). Shared developmental programme strongly constrains beak shape diversity in songbirds. *Nat. Commun.* 5, 3700. 10.1038/ncomms4700 24739280

[DEV196253C16] Geitmann, A. and Ortega, J. K. (2009). Mechanics and modeling of plant cell growth. *Trends Plant Sci.* 14, 467-478. 10.1016/j.tplants.2009.07.006 19717328

[DEV196253C17] Goh, T., Toyokura, K., Wells, D. M., Swarup, K., Yamamoto, M., Mimura, T., Weijers, D., Fukaki, H., Laplaze, L., Bennett, M. J. et al. (2016). Quiescent center initiation in the Arabidopsis lateral root primordia is dependent on the SCARECROW transcription factor. *Development* 143, 3363-3371. 10.1242/dev.135319 27510971

[DEV196253C18] Hamamoto, L., Hawes, M. C. and Rost, T. L. (2006). The production and release of living root cap border cells is a function of root apical meristem type in dicotyledonous angiosperm plants. *Ann Bot* 97, 917-923. 10.1093/aob/mcj602 16488922 PMC2803423

[DEV196253C19] Hamant, O., Heisler, M. G., Jonsson, H., Krupinski, P., Uyttewaal, M., Bokov, P., Corson, F., Sahlin, P., Boudaoud, A., Meyerowitz, E. M. et al. (2008). Developmental patterning by mechanical signals in Arabidopsis. *Science* 322, 1650-1655. 10.1126/science.1165594 19074340

[DEV196253C20] Handy, R. L. (1973). The igloo and the natural bridge as ultimate structures. *Arctic* 26, 276-281. 10.14430/arctic2926

[DEV196253C21] Heimsch, C. and Seago Jr., J. L. (2008). Organization of the root apical meristem in angiosperms. *Am. J. Bot.* 95, 1-21. 10.3732/ajb.95.1.1 21632311

[DEV196253C22] Hejnowicz, Z. (1984). Trajectories of principal directions of growth, natural coordinate system in growing plant organ. *Acta Soc. Bot. Pol.* 53, 29-42. 10.5586/asbp.1984.004

[DEV196253C23] Hervieux, N., Tsugawa, S., Fruleux, A., Dumond, M., Routier-Kierzkowska, A. L., Komatsuzaki, T., Boudaoud, A., Larkin, J. C., Smith, R. S., Li, C. B. et al. (2017). Mechanical shielding of rapidly growing cells buffers growth heterogeneity and contributes to organ shape reproducibility. *Curr. Biol.* 27, 3468-3479.e3464. 29129534 10.1016/j.cub.2017.10.033

[DEV196253C24] Heyman, J. (1998). Hooke's cubico–parabolical conoid. *Notes Rec. R Soc. Lond.* 52, 39-50. 10.1098/rsnr.1998.0033

[DEV196253C25] Hirota, A., Kato, T., Fukaki, H., Aida, M. and Tasaka, M. (2007). The auxin-regulated AP2/EREBP Gene PUCHI is required for morphogenesis in the early lateral root primordium of arabidopsis. *Plant Cell* 19, 2156-2168. 10.1105/tpc.107.050674 17630277 PMC1955702

[DEV196253C26] Honda, H. (1983). Geometrical models for cells in tissues. *Int. Rev. Cytol.* 81, 191-248. 10.1016/S0074-7696(08)62339-6 6347934

[DEV196253C27] Hong, L., Dumond, M., Tsugawa, S., Sapala, A., Routier-Kierzkowska, A.-L., Zhou, Y., Chen, C., Kiss, A., Zhu, M., Hamant, O. et al. (2016). Variable cell growth yields reproducible organ development through spatiotemporal averaging. *Dev. Cell* 38, 15-32. 10.1016/j.devcel.2016.06.016 27404356

[DEV196253C28] Hong, L., Dumond, M., Zhu, M., Tsugawa, S., Li, C. B., Boudaoud, A., Hamant, O. and Roeder, A. H. K. (2018). Heterogeneity and robustness in plant morphogenesis: from cells to organs. *Annu. Rev. Plant Biol.* 69, 469-495. 10.1146/annurev-arplant-042817-040517 29505739

[DEV196253C29] Houle, D., Bolstad, G. H., van der Linde, K. and Hansen, T. F. (2017). Mutation predicts 40 million years of fly wing evolution. *Nature* 548, 447-450. 10.1038/nature23473 28792935

[DEV196253C30] Huang, L. and Schiefelbein, J. (2015). Conserved gene expression programs in developing roots from diverse plants. *Plant Cell* 27, 2119-2132. 10.1105/tpc.15.00328 26265761 PMC4568505

[DEV196253C31] Kumpf, R. P. and Nowack, M. K. (2015). The root cap: a short story of life and death. *J. Exp. Bot.* 66, 5651-5662. 10.1093/jxb/erv295 26068468

[DEV196253C32] Kurihara, D., Mizuta, Y., Sato, Y. and Higashiyama, T. (2015). ClearSee: a rapid optical clearing reagent for whole-plant fluorescence imaging. *Development* 142, 4168-4179. 10.1242/dev.127613 26493404 PMC4712841

[DEV196253C33] Lavenus, J., Goh, T., Roberts, I., Guyomarc'h, S., Lucas, M., De Smet, I., Fukaki, H., Beeckman, T., Bennett, M. and Laplaze, L. (2013). Lateral root development in Arabidopsis: fifty shades of auxin. *Trends Plant Sci.* 18, 450-458. 10.1016/j.tplants.2013.04.006 23701908

[DEV196253C34] Lavenus, J., Goh, T., Guyomarc'h, S., Hill, K., Lucas, M., Voss, U., Kenobi, K., Wilson, M. H., Farcot, E., Hagen, G. et al. (2015). Inference of the Arabidopsis lateral root gene regulatory network suggests a bifurcation mechanism that defines primordia flanking and central zones. *Plant Cell* 27, 1368-1388. 10.1105/tpc.114.132993 25944102 PMC4456640

[DEV196253C35] Leiboff, S., Li, X., Hu, H. C., Todt, N., Yang, J., Li, X., Yu, X., Muehlbauer, G. J., Timmermans, M. C., Yu, J. et al. (2015). Genetic control of morphometric diversity in the maize shoot apical meristem. *Nat. Commun.* 6, 8974. 10.1038/ncomms9974 26584889 PMC4673881

[DEV196253C36] Leiboff, S., DeAllie, C. K. and Scanlon, M. J. (2016). Modeling the morphometric evolution of the maize shoot apical meristem. *Front. Plant Sci.* 7, 1651. 10.3389/fpls.2016.01651 27867389 PMC5095129

[DEV196253C37] Le Roy, C., Debat, V. and Llaurens, V. (2019). Adaptive evolution of butterfly wing shape: from morphology to behaviour. *Biol. Rev. Camb. Philos. Soc.* 94, 1261-1281. 10.1111/brv.12500 30793489

[DEV196253C38] Lockhart, J. A. (1965). An analysis of irreversible plant cell elongation. *J. Theor. Biol.* 8, 264-275. 10.1016/0022-5193(65)90077-9 5876240

[DEV196253C39] Lockwood, E. H. (1961). *Book of Curves*. Cambridge: Cambridge University Press.

[DEV196253C40] Lucas, M., Kenobi, K., Wangenheim, D. V., Voβ, U., Swarup, K., Smet, I. D., Damme, D. V., Lawrence, T., Péret, B., Moscardi, E. et al. (2013). Lateral root morphogenesis is dependent on the mechanical properties of the overlaying tissues. *Proc. Natl. Acad. Sci. U.S.A.* 110, 5229-5234. 10.1073/pnas.1210807110 23479644 PMC3612681

[DEV196253C41] Maugarny-Cales, A. and Laufs, P. (2018). Getting leaves into shape: a molecular, cellular, environmental and evolutionary view. *Development* 145, dev161646. 10.1242/dev.161646 29991476

[DEV196253C42] Mellor, N., Vaughan-Hirsch, J., Kümpers, B. M. C., Help-Rinta-Rahko, H., Miyashima, S., Mähönen, A. P., Campilho, A., King, J. R. and Bishopp, A. (2019). A core mechanism for specifying root vascular patterning can replicate the anatomical variation seen in diverse plant species. *Development* 146, dev172411. 10.1242/dev.172411 30858228 PMC6451317

[DEV196253C43] Mishra, A. K., Tramacere, F., Guarino, R., Pugno, N. M. and Mazzolai, B. (2018). A study on plant root apex morphology as a model for soft robots moving in soil. *PLoS ONE* 13, e0197411. 10.1371/journal.pone.0197411 29874267 PMC5991344

[DEV196253C44] Moré, J. J. (1978). The Levenberg-Marquardt algorithm: implementation and theory (ed. G. A. Watson), pp. 105-116: Springer.

[DEV196253C45] Musielak, T. J., Schenkel, L., Kolb, M., Henschen, A. and Bayer, M. (2015). A simple and versatile cell wall staining protocol to study plant reproduction. *Plant Reproduction* 28, 161-169. 10.1007/s00497-015-0267-1 26454832 PMC4623088

[DEV196253C46] Nakielski, J. and Lipowczan, M. (2013). Spatial and directional variation of growth rates in Arabidopsis root apex: a modelling study. *PLOS ONE* 8, e84337. 10.1371/journal.pone.0084337 24367654 PMC3867472

[DEV196253C47] Niklas, K. J. (1994). *Plant Allometry: the Scaling of form and Process*. Chicago: University of Chicago Press.

[DEV196253C48] Norman, J. M. V., Xuan, W., Beeckman, T. and Benfey, P. N. (2013). To branch or not to branch: the role of pre-patterning in lateral root formation. *Development* 140, 4301-4310. 10.1242/dev.090548 24130327 PMC4007709

[DEV196253C49] Okada, K. and Shimura, Y. (1992). Mutational analysis of root gravitropism and phototropism of Arabidopsis thaliana seedlings. *Funct. Plant Biol.* 19, 439-448. 10.1071/PP9920439

[DEV196253C50] Ortega, J. K. (1985). Augmented growth equation for cell wall expansion. *Plant Physiol.* 79, 318-320. 10.1104/pp.79.1.318 16664396 PMC1074876

[DEV196253C51] Petricka, J. J., Winter, C. M. and Benfey, P. N. (2012). Control of Arabidopsis root development. *Annu. Rev. Plant Biol.* 63, 563-590. 10.1146/annurev-arplant-042811-105501 22404466 PMC3646660

[DEV196253C52] Roue, J., Chauvet, H., Brunel-Michac, N., Bizet, F., Moulia, B., Badel, E. and Legue, V. (2020). Root cap size and shape influence responses to the physical strength of the growth medium in Arabidopsis thaliana primary roots. *J. Exp. Bot.* 71, 126-137. 10.1093/jxb/erz418 31682268

[DEV196253C53] Sakamoto, Y., Ishiguro, M. and Kitagawa, G. (1986). *Akaike Information Criterion Statistics*. Tokyo, MA: KTK Scientific Publishers; D. Reidel; Sold and distributed in the U.S.A. and Canada by Kluwer Academic Publishers.

[DEV196253C54] Salcedo, M. K., Hoffmann, J., Donoughe, S. and Mahadevan, L. (2019). Computational analysis of size, shape and structure of insect wings. *Biol Open* 8, bio040774. 10.1242/bio.040774 31628142 PMC6826288

[DEV196253C55] Schmidt-Nielsen, K. (1984). *Scaling, Why is Animal Size so Important?* Cambridge; New York: Cambridge University Press.

[DEV196253C56] Szymanowska-Pułka, J., Potocka, I., Karczewski, J., Jiang, K., Nakielski, J. and Feldman, L. J. (2012). Principal growth directions in development of the lateral root in Arabidopsis thaliana. *Ann Bot* 110, 491-501. 10.1093/aob/mcs129 22700942 PMC3394657

[DEV196253C57] ten Hove, C. A., Lu, K.-J. and Weijers, D. (2015). Building a plant: cell fate specification in the early Arabidopsis embryo. *Development* 142, 420-430. 10.1242/dev.111500 25605778

[DEV196253C58] Thompson, D. A. W. (1917). *On Growth and Form*: Cambridge University Press.

[DEV196253C59] Timoshenko, S. P. and Gere, J. M. (1961). *Theory of Elastic Stability*: McGraw-Hill.

[DEV196253C60] Torres-Martinez, H. H., Rodriguez-Alonso, G., Shishkova, S. and Dubrovsky, J. G. (2019). Lateral root primordium morphogenesis in angiosperms. *Front Plant Sci* 10, 206. 10.3389/fpls.2019.00206 30941149 PMC6433717

[DEV196253C61] Trinh, D.-C., Lavenus, J., Goh, T., Boutté, Y., Drogue, Q., Vaissayre, V., Tellier, F., Lucas, M., Voß, U., Gantet, P. et al. (2019). PUCHI regulates very long chain fatty acid biosynthesis during lateral root and callus formation. *Proc. Natl. Acad. Sci. USA* 116, 14325-14330. 10.1073/pnas.1906300116 31235573 PMC6628829

[DEV196253C62] Tsukaya, H. (2018). Leaf shape diversity with an emphasis on leaf contour variation, developmental background, and adaptation. *Semin. Cell Dev. Biol.* 79, 48-57. 10.1016/j.semcdb.2017.11.035 29199138

[DEV196253C63] Uyttewaal, M., Burian, A., Alim, K., Landrein, B., Borowska-Wykret, D., Dedieu, A., Peaucelle, A., Ludynia, M., Traas, J., Boudaoud, A. et al. (2012). Mechanical stress acts via katanin to amplify differences in growth rate between adjacent cells in Arabidopsis. *Cell* 149, 439-451. 10.1016/j.cell.2012.02.048 22500806

[DEV196253C64] Van Damme, D., Rybel, B. D., Gudesblat, G., Demidov, D., Grunewald, W., Smet, I. D., Houben, A., Beeckman, T. and Russinova, E. (2011). Arabidopsis α aurora kinases function in formative cell division plane orientation. *Plant Cell* 23, 4013-4024. 10.1105/tpc.111.089565 22045917 PMC3246319

[DEV196253C65] Vermeer, J. E. M., Wangenheim, D. V., Barberon, M., Lee, Y., Stelzer, E. H. K., Maizel, A. and Geldner, N. (2014). A spatial accommodation by neighboring cells is required for organ initiation in arabidopsis. *Science* 343, 178-183. 10.1126/science.1245871 24408432

[DEV196253C66] von Wangenheim, D., Fangerau, J., Schmitz, A., Smith, R. S., Leitte, H., Stelzer, E. H. K. and Maizel, A. (2016). Rules and self-organizing properties of post-embryonic plant organ cell division patterns. *Curr. Biol.* 26, 439-449. 10.1016/j.cub.2015.12.047 26832441

[DEV196253C67] Wang, X. and Clarke, J. A. (2015). The evolution of avian wing shape and previously unrecognized trends in covert feathering. *Proc. Biol. Sci.* 282, 20151935. 10.1098/rspb.2015.1935 26446812 PMC4614784

