# Supplementary material for: Tissue growth constrains root organ outlines into an isometrically scalable shape
Source: Development. 2021 Feb 26;148(4):dev196253. doi: 10.1242/dev.196253 (PMC7929931; doi:10.1242/dev.196253)
Supplement: Supplementary information [file develop-148-196253-s1.pdf]

Supporting Material

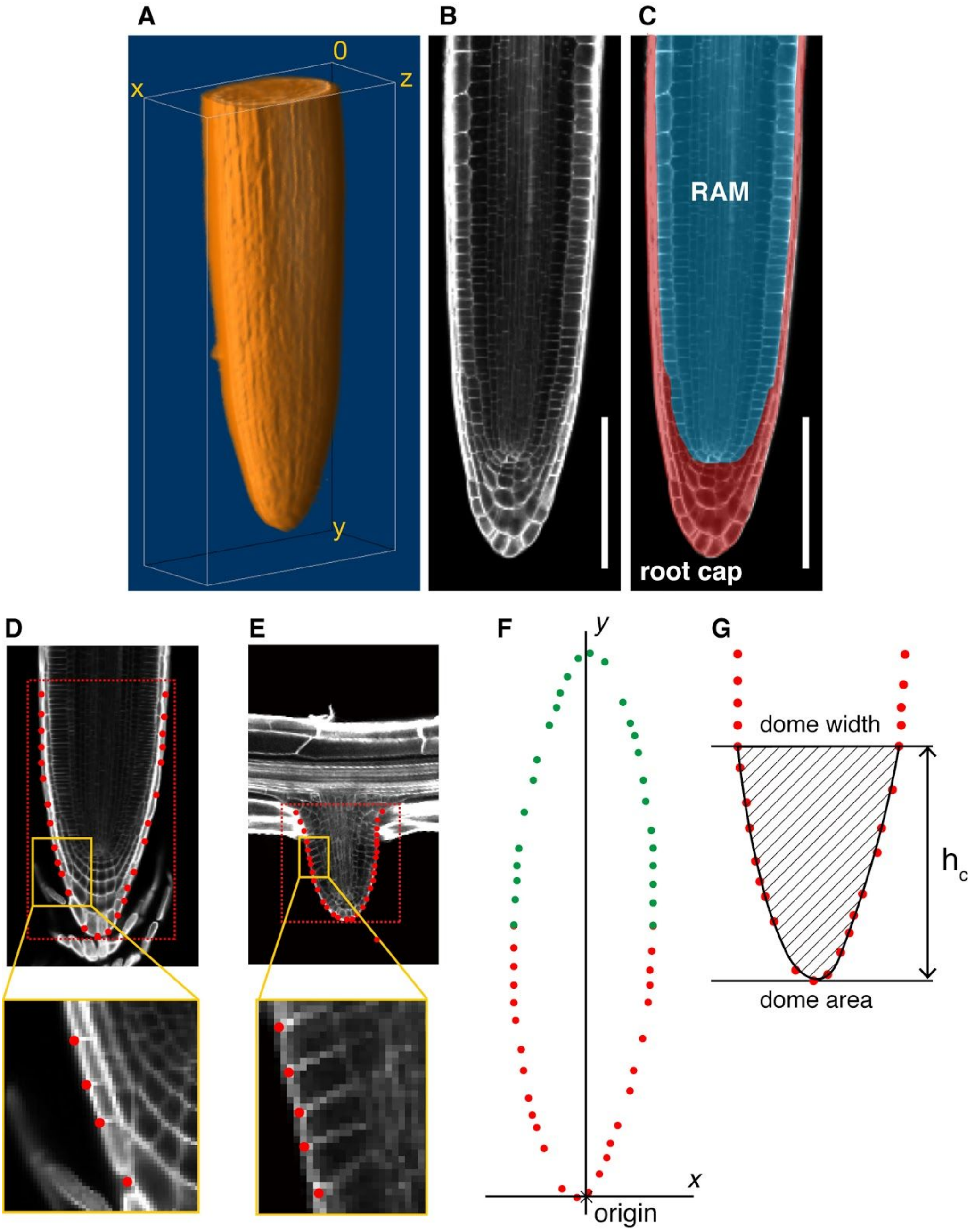

Fig. S1

Determination of root dome outlines and unification of the coordinate system.

(A) 3D view of a mature LR (lateral root).

Development • Supplementary information

(B, C) Raw image of a vertical section of a mature LR (B). The root tip is composed of RAM (root apical meristem; blue region) and a root cap (red region) (C). Scale bars indicate 100 µm.

(D, E) Root tip outlines of a PR (primary root; D) and an LR (E) were determined from the cell junction positions on the dome outlines (red points, enlarged figure in bottom panel). Red dotted boxes denote the region of interest, which ranged from the root tip to the boundary between the proliferation and elongation zone (D) and to the vascular cells of parent roots (E).

(F) Unification of the coordinate system (see Material and Methods). Red points indicate the original positions on the root tip outline, and green points indicate the points duplicated and turned by 180 degree. The origin of x- and y-coordinates were determined as the mean of x of all points and the minimal y of the original points.

(G) Illustration of how to measure the dome width and area (shaded region) up to the indicated height h_c from the root tip.

Development • Supplementary information

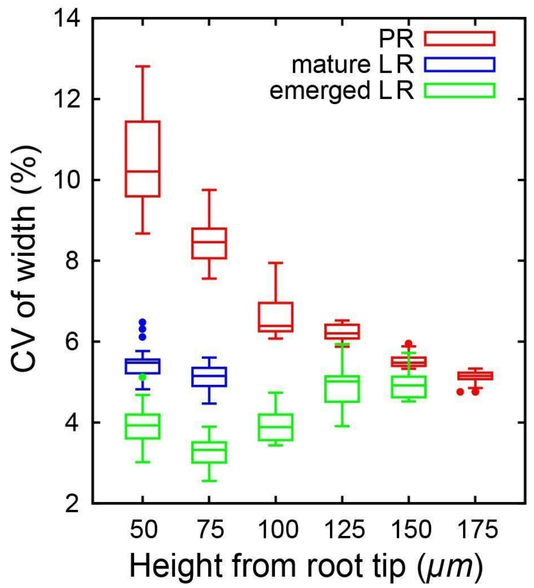

Fig. S2

Size reproducibility of root tip width.

Root tip dome width measured on the median longitudinal section up to the indicated heights from the root tip. Root tip width measured on the median longitudinal section up to the indicated height from the dome tip (25 µm steps). The size reproducibility is indicated by the coefficient of variation (CV (%) = (SD of width) x 100 / (mean of width)). The lower and upper hinges, the middle lines and the error bars of box plots represent the 25th, 75th, and 50th percentiles, and SD, respectively. Data sets were identical to those of Fig. 1B-E.

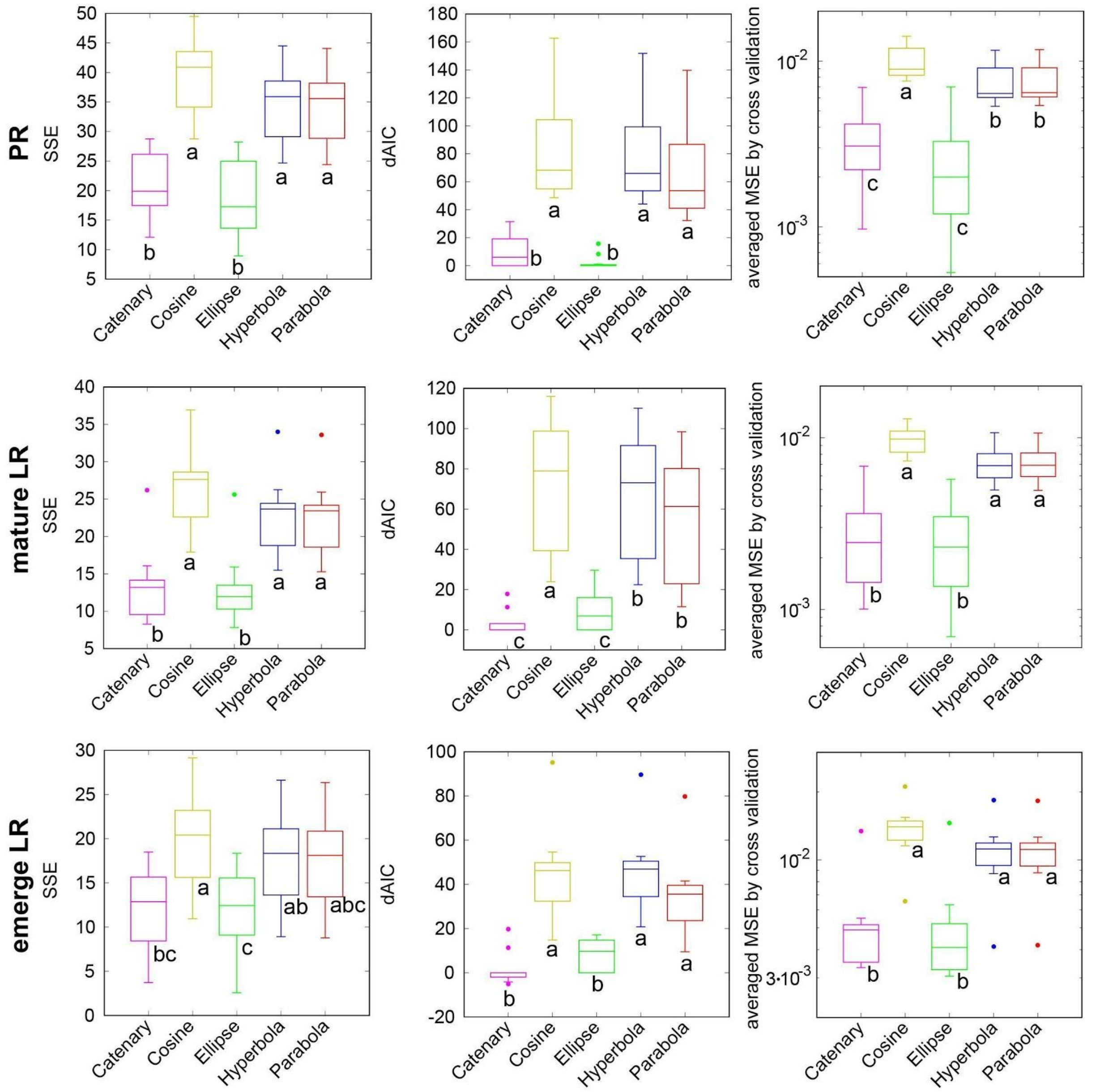

Fig. S3

Statistical model selection of best-fitting model of the root tip outlines of *Arabidopsis* using SSE, ΔAIC (Akaike information criterion) and average MSE (mean squared error).

SSE (left panels), ΔAIC (central panels), and average MSE by cross validation (right panels) between the dome outlines and the five model functions. SSE and average MSE for PRs were identical with Fig. 2C, E, respectively. The lower and upper hinges, the middle lines, and the error bars of box plots represent the 25th, 75th, and 50th percentiles, and SD, respectively. Sample numbers are n = 12 (PR), n = 11 (mature LR), and n = 12 (emerged LR). See Material and Methods for definition of SSE, ΔAIC and MSE. Different letters (a, b, c) denote statistically significant differences ($P < 0.05$) among means by Tukey's honestly significant difference (HSD) test.

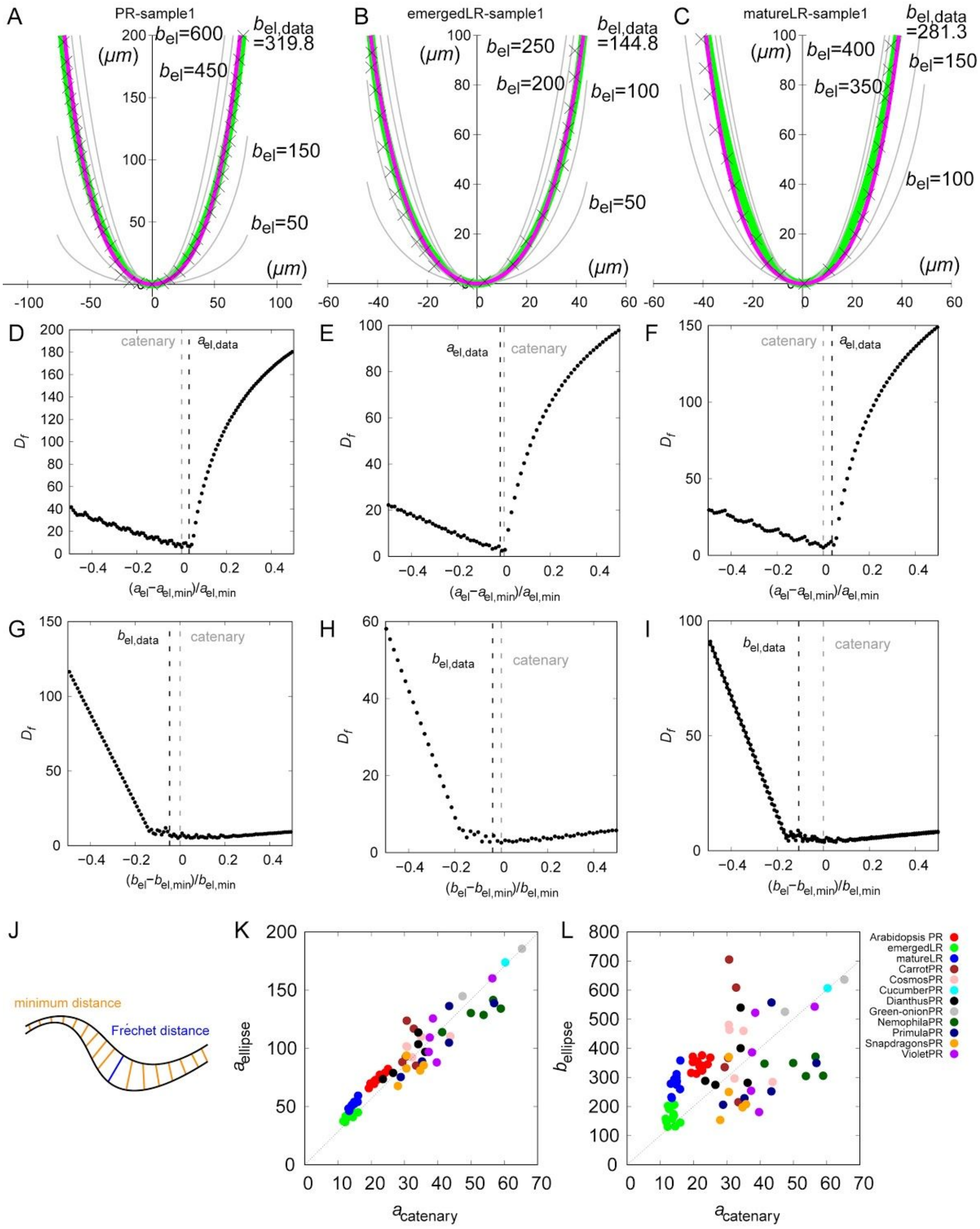

Fig. S4

Quantitative comparison of the catenary and ellipse functions

(A-C) The fitted function of a catenary (magenta) and an ellipse (green) to a sample of a PR (A), an emerged LR (B) and a matured LR (C). Grey lines indicate ellipses ($y = b_{ellipse} - b_{ellipse}\sqrt{1 - x^2/{a_{ellipse}}^2}$) with different values of the ellipse parameter $b_{ellipse}$

Development • Supplementary information

(abbreviated as b_{el}) given the fitted value of the other ellipse parameter $a_{ellipse}$ ($a_{ellipse}$= 78.0 [A], = 45.0 [B] and = 50.1 [C]; abbreviated as a_{el}), demonstrating that the fitted ellipse is apparently closest to the fitted catenary among the ellipses. Data points (X) represent root tip outlines identical to those in Fig. 1A.

(D-J) We quantitatively evaluated the closeness using the bottleneck distance between two different curves (J), known as the Fréchet distance given by $D_f = inf_{\alpha,\beta} max_{t\in[0,1]} \{d(catenary(\alpha(t)), ellipse(\beta(t)))\}$, where d denotes distance function; α and β denote reparametrization of $[0, 1]$ to a catenary and an ellipse, respectively, within the range of the plant root sample width. Fréchet distance was numerically measured by discretizing t into 200 equi-spaced samples. We revealed that the fitted ellipse (black dashed line) was almost closest to the fitted catenary curve (grey dashed line) among any ellipses given the fitted value of $b_{ellipse}$ (abbreviated as $b_{el.data}$; D-F) or $a_{ellipse}$ (abbreviated as $a_{el.data}$; G-I), for all samples of PRs (D, G), emerged LRs (E, H) and matured LRs (F, I). $a_{el.min}$ and $b_{el.min}$ denote the parameters of the closest ellipse indicating the global minimum of Fréchet distance, whereas the multiple local minima and maxima in the order of ± 1 are due to numerical errors.

(K, L) The fitted values of $a_{ellipse}$ (K) and $b_{ellipse}$ (L) were proportional to the fitted catenary parameter $a_{catenary}$ among PR and LR samples, i.e., $a_{ellipse} = 2.84a_{catenary}$ and $b_{ellipse} = 10.0a_{catenary}$, respectively (grey dotted line). This proportionality enabled us to parametrize the ellipse by $a_{catenary}$ alone via substituting the proportionalities: $y = 10.0a_{catenary}\sqrt{1-\left(\frac{x}{2.84a_{catenary}}\right)^2}$. We referred to this function as the catenary-closest ellipse. By scaling of both x- and y-coordinates with $a_{catenary}$, any catenary-closest ellipses commonly converge to a unique function ($Y = 10.0\sqrt{1-\left(\frac{X}{2.84}\right)^2}$, $X = x/a_{catenary}$, $Y = y/a_{catenary}$).

Development • Supplementary information

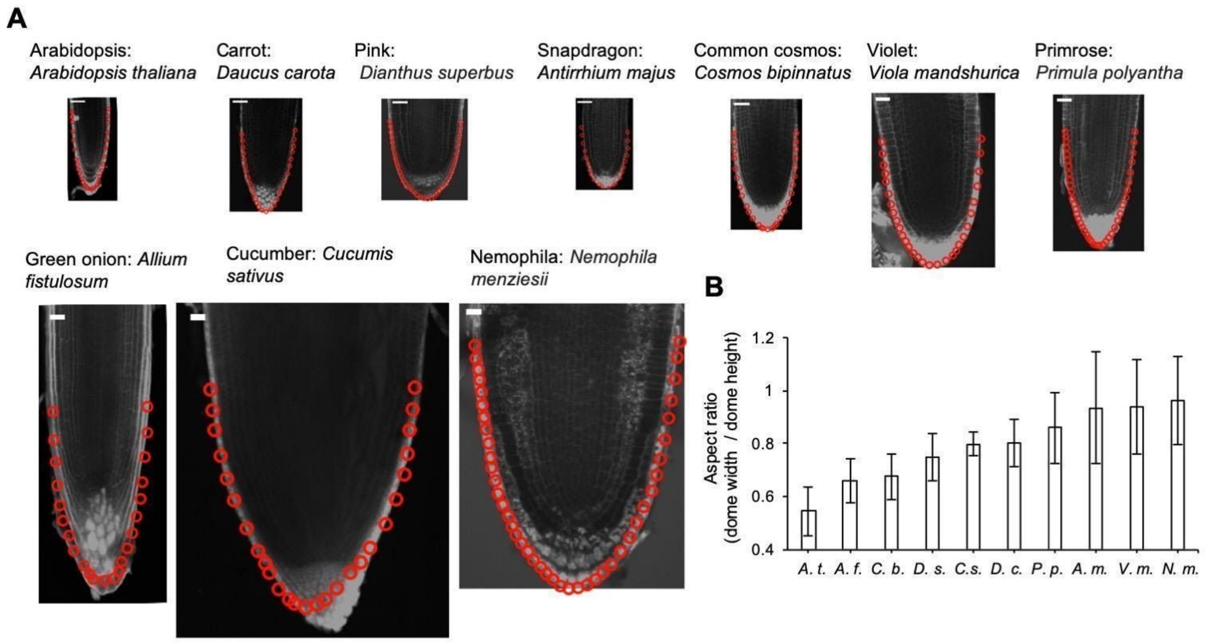

C

Initial of species name	*A. t.*	*A. f.*	*C. b.*	*D. s.*	*C. s.*	*D. c.*	*P. p.*	*A. m.*	*V. m.*	*N. m.*
Meristem type	Close	Open	Close	Close	Open	Close	Close	Close	Close	Close
Grand-tissue cell layers	2	2	3	2	5	3	3	2	2	4

Fig. S5

PR tip outlines in nine angiosperm species.

(A) Root tip outline (red circles) of each species. Scale bars indicate 50 µm.

(B) The aspect ratio (dome width / dome height) of the root tip for each species (n = 5 for each species).

(C) The cellular organization of root tips among species. The meristem types and the number of ground-tissue cell layers appeared to be species-dependent.

Development • Supplementary information

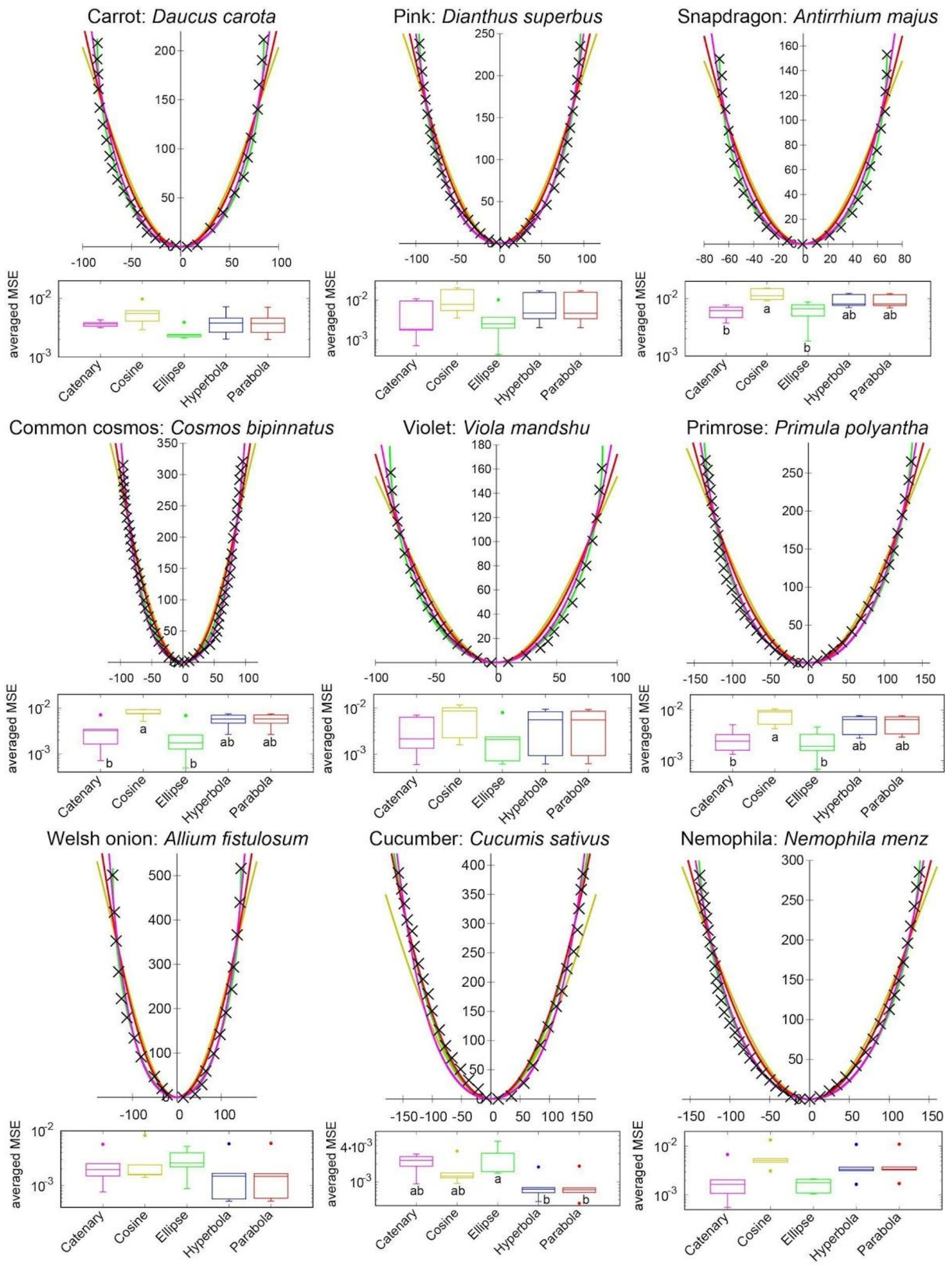

Fig. S6

Average MSE of PR tip shape in nine angiosperm species.

The average MSE from a cross validation test with the five functions for each species (n = 5 for each species). Different letters (a, b ,c) denote statistically significant differences ($P < 0.05$) among means by Tukey's honestly significant difference (HSD) test.

Development • Supplementary information

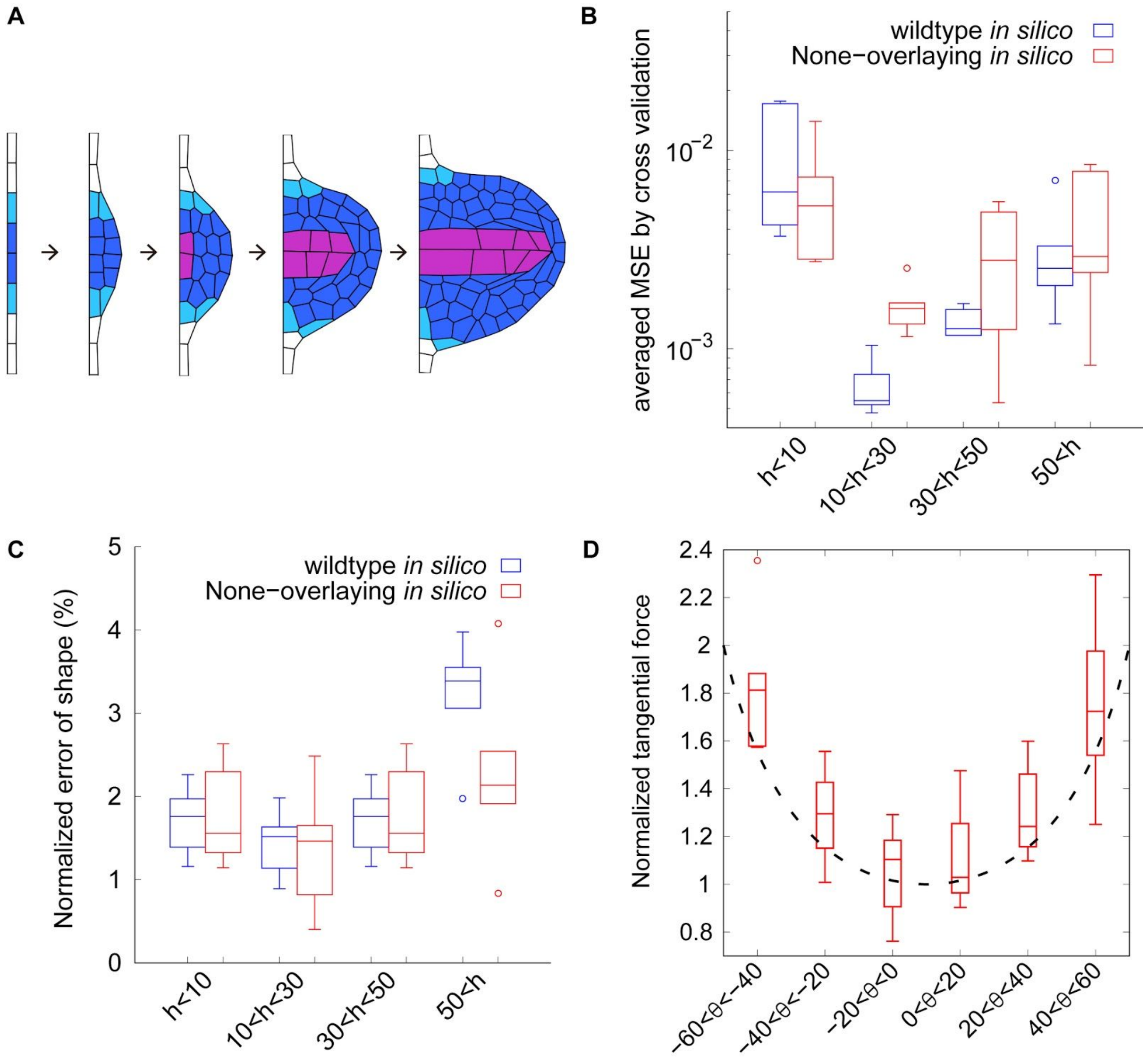

Fig. S7

LRP development *in silico.*

(A) Simulation without a giant imaginal cell (none-overlaying cell model).

(B, C) average MSE from the cross validation test with a catenary curve (B; Eq. 12) and the shape reproducibility indicator (C; Eq. 9) of tip outline during simulations (*in silico*) of the wild type (blue; identical with Fig. 4C, red) and the non-overlaying cell (red; n = 5 for each dome height range, h < 10, 10 < h < 30 and 30 < h < 50) models.

(D) Tangential force on the LRP surface. The magnitude of tangential force (black arrow in Fig. 4I bottom panel) after cell expansion. Vertex model simulations (box plot) and theoretical prediction based on a catenary-curved chain ($T/T_0 = 1/\cos\theta$ in Fig. 4H; dashed line). The force of each cell on the outline (red arrow in Fig. 4I, upper panel) was normalized by that at the dome tip and plotted as a function of θ of the x-coordinate of the dome (Fig. 4H). The lower and upper hinges, and the middle lines of box plots represent the 25th, 75th, and 50th percentiles, respectively. The error bars denote the SD of five independent simulations. Sample sets were identical to those of Fig. 4I.

Development • Supplementary information

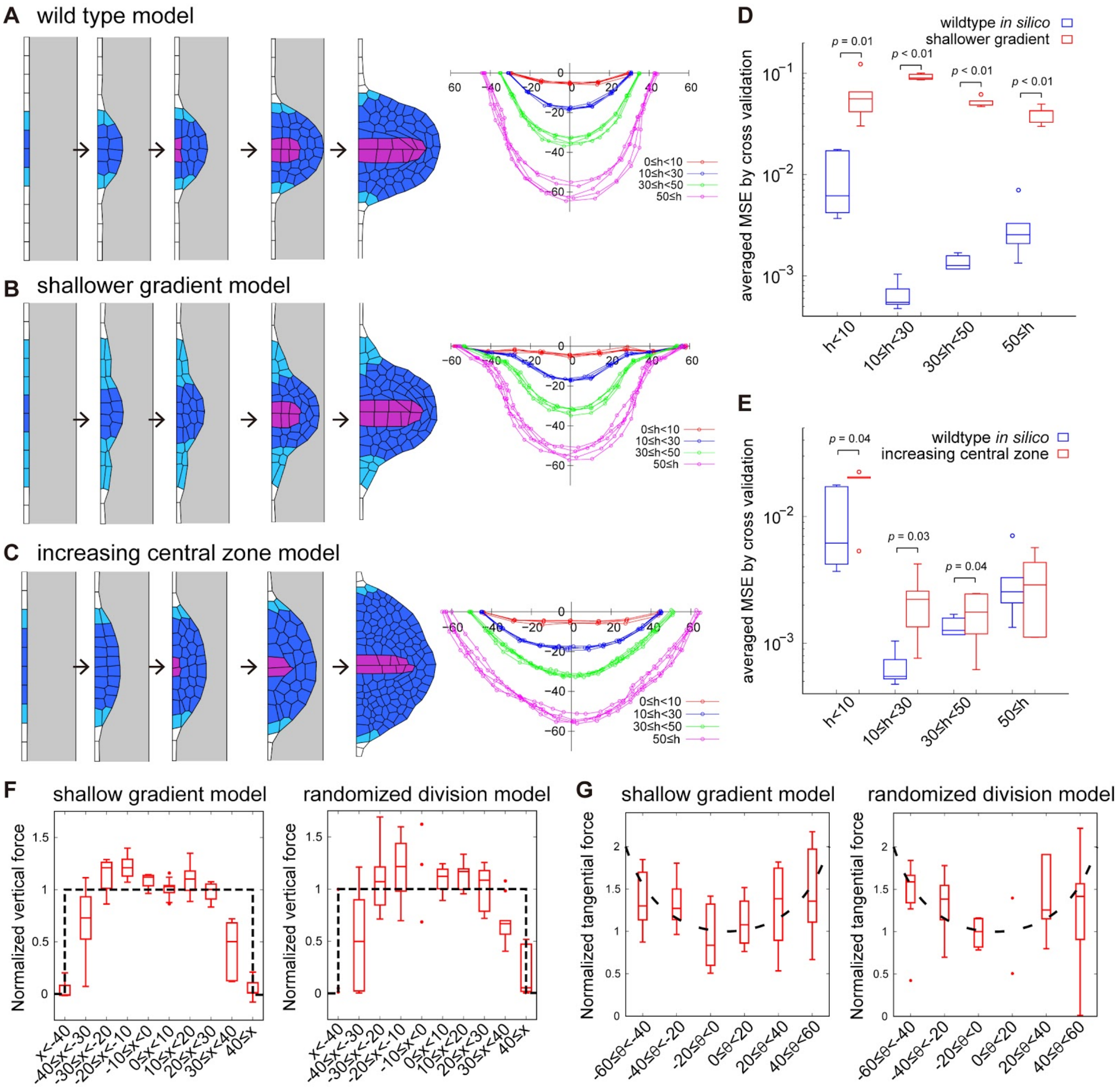

Fig. S8

Tissue growth rules affects its dome shape

(A-C) Developmental time course of vertex model simulations (left) and the root tip outlines (right) of the wildtype model (A; Fig. 4), the shallower gradient model (B) and the increasing central zone model (C). Color coding of cells (left) and outlines (right) are identical with that of Fig. 5A and B, respectively. **(D, E)** The average MSE from the cross validation test with a catenary curve (n = 5 for each dome height range, $h < 10$, $10 \leq h < 30$ and $30 \leq h < 50$) in wild type model (A), the shallower gradient (by increasing flanking region, light blue cells) model (B) and the increasing central region (dark blue cells) model (C). **(F, G)** The magnitude of vertical force (F) and tangential force (G) normalized by its spatial average over the dividing zone (dark blue and light blue cells in the right panel in Fig. 5A, F) plotted as a function of x-coordinate along the dome width in the shallow gradient model model (left panel) and the randomized division model (right panel). The lower and upper hinges, and the middle lines of box plots in D, E, F and G represent the 25th, 75th, and 50th percentiles, respectively. The error bars denote the SD of five independent simulations

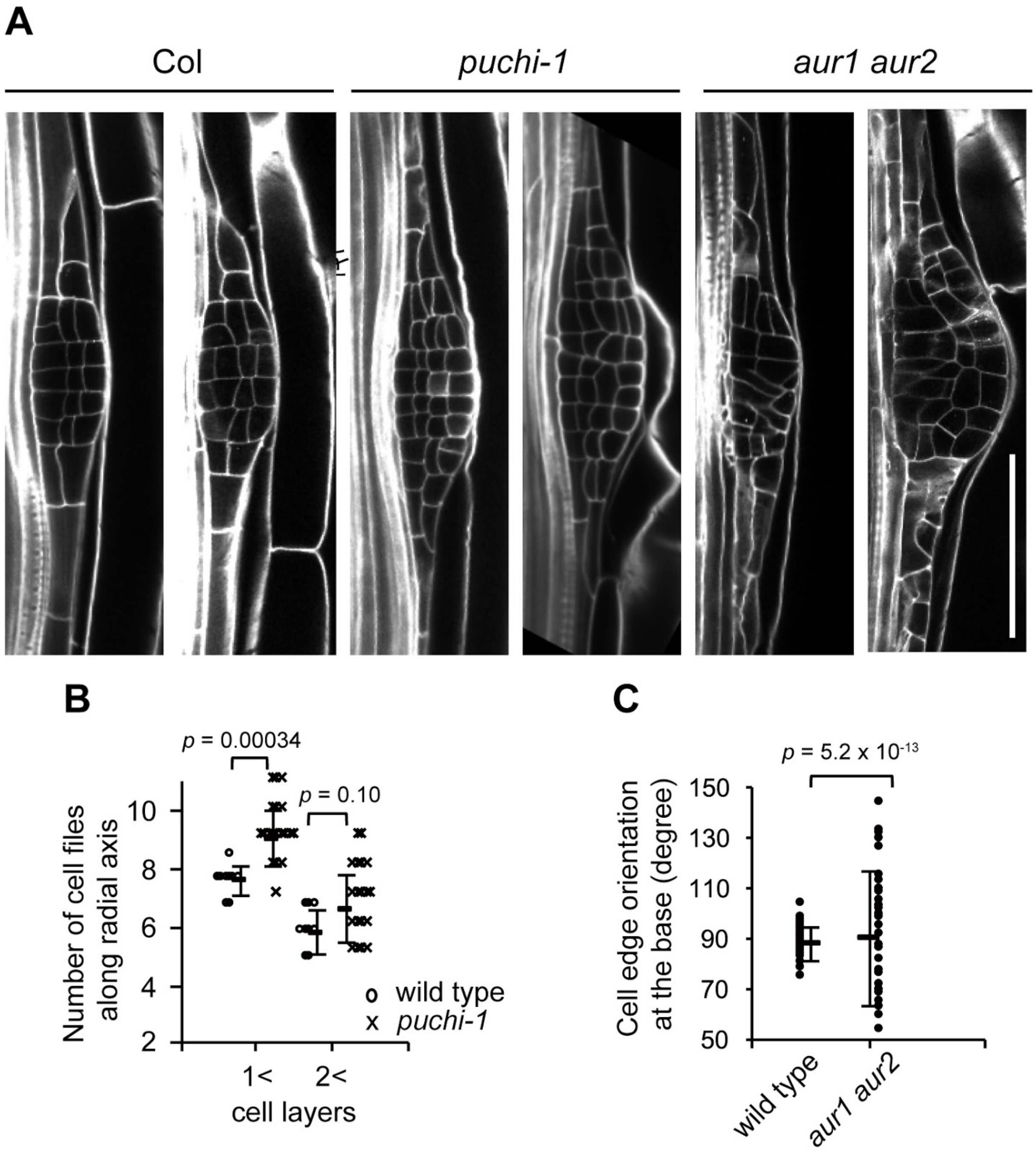

Fig. S9

Cell division defects of *puchi-1* and *aur1 aur2 in vivo*.

(A) Stage IV LRPs of Col, *puchi-1* and *aur1 aur2*. Scale bar = 50 µm.

(B) The number of cell files with more than one or two cell layers in the wild type (left, n = 5) and the *puchi-1* mutant (right, n = 17) at stage IV.

(C) The orientation of cell division θ in the wild type (left, n = 5) and the *aur1 aur2* mutant (right, n = 5) at stage IV. A *t*-test was performed after confirming a normal distribution by Kolmogorov-Smirnov test. The error bars in B and C indicate SD.

Development • Supplementary information

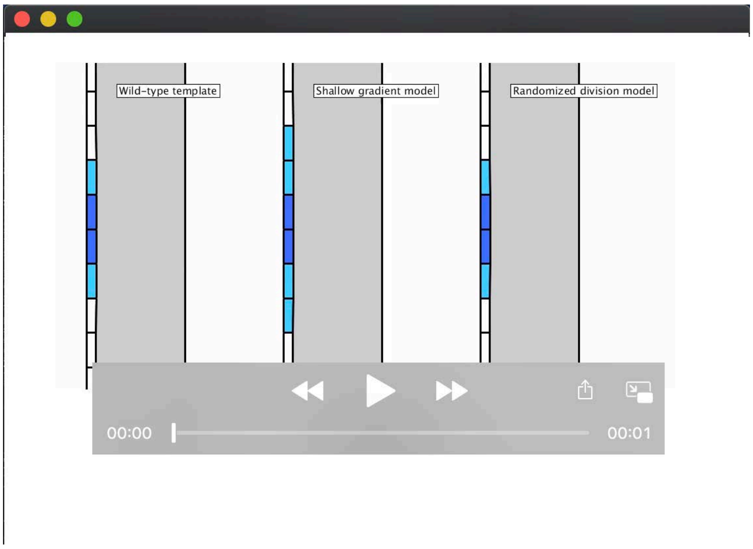

Movie 1.
Simulation of dome formation from stage I to VII under the wild type template (left, Fig. 4E), shallow gradient in cell division rate (center, Fig. 5A), and randomization in cell division order (right, Fig. 5F).
