# Supplementary material for: Tissue growth constrains root organ outlines into an isometrically scalable shape
Source: Development. 2021 Feb 26;148(4):dev196253. doi: 10.1242/dev.196253 (PMC7929931; doi:10.1242/dev.196253)
Supplement: Reviewer comments [file develop-148-196253_review_history.pdf]

Tissue growth constrains root organ outlines into an isometrically scalable shape

Motohiro Fujiwara, Tatsuaki Goh, Satoru Tsugawa, Keiji Nakajima, Hidehiro Fukaki and Koichi Fujimoto
DOI: 10.1242/dev.196253

Editor: Yka Helariutta

Review timeline

Original submission:	23 August 2020
Editorial decision:	28 September 2020
First revision received:	18 December 2020
Accepted:	11 January 2021

Original submission

First decision letter

MS ID#: DEVELOP/2020/196253

MS TITLE: Tissue growth constrains root organ outlines into an isometrically scalable shape

AUTHORS: Motohiro Fujiwara, Tatsuaki Goh, Satoru Tsugawa, Keiji Nakajima, Hidehiro Fukaki, and Koichi Fujimoto

I have now received all the referees reports on the above manuscript, and have reached a decision. The referees' comments are appended below, or you can access them online: please go to BenchPress and click on the 'Manuscripts with Decisions' queue in the Author Area.

The overall evaluation is positive and we would like to publish a revised manuscript in Development, provided that the referees' comments can be satisfactorily addressed. Please attend to all of the reviewers' comments in your revised manuscript and detail them in your point-by-point response. If you do not agree with any of their criticisms or suggestions explain clearly why this is so.

We are aware that you may currently be unable to access the lab to undertake experimental revisions. If it would be helpful, we encourage you to contact us to discuss your revision in greater detail. Please send us a point-by-point response indicating where you are able to address concerns raised (either experimentally or by changes to the text) and where you will not be able to do so within the normal timeframe of a revision. We will then provide further guidance. Please also note that we are happy to extend revision timeframes as necessary.

Reviewer 1

Advance summary and potential significance to field

The shape of the plant root tip has been previously reported to match that of an ellipse. In this work the authors report that this structureÂ’s curvature also matches a catenary arch, representing the strongest curved structure. These geometric matches are reported for a series of species.

© 2021. Published by The Company of Biologists under the terms of the Creative Commons Attribution License (https://creativecommons.org/licenses/by/4.0/).

Comments for the author

The implication of the catenary arch is that the shape of the root is mechanically optimized to penetrate soil impedance.
The authors proceed to generate models describing the generative processes leading to this curve. The PUCHI and AURORA KINASE mutants are shown to have different LRP shapes, and a plausible mechanism underpinning these geometric differences is proposed.
This represents an interesting and well-presented manuscript for which I have no major comments or objections, with the exception of the functional implications of the thesis.
If the catenary curve is indeed a mechanically optimised shape in the context of roots penetrating soil, this remains to be demonstrated. The authors have the necessary genetic materials in the form of the wild-type, PUCHI and AURORA mutants to explore the mechanical properties each of these root shapes exhibit in the face of mechanical impedance.
It remains important to demonstrate this point as the vector forces applied to an arch on a bridge are different to that of a drill bit penetrating a material. The latter example more closely represents the process of roots entering into soil. The addition of these in vivo mechanical experiments would significantly enhance the conclusions of this manuscript.

Minor points relate to the abstract where the use of mutants and the computational simulations merit metioning.

<u>Reviewer 2</u>

Advance summary and potential significance to field

This study provides interesting insights into the developmental constraints shaping the geometry of Root Apical Meristems (RAMs). It shows that RAMs in Arabidopsis and 9 other species can be well approximated by a catenary curve. This allows root tip geometry to be characterized by a single shape parameter alpha, which is roughly the inverse of curvature. The ability to capture overall wild-type RAM geometry as a single interpretable shape parameter is noteworthy and seems useful for future quantitative studies, as is the finding that the various RAMs analyzed exhibit isometric scaling.

Although other functions approximate RAMs similarly (e.g. ellipses), catenary curves have noteworthy mechanical properties. Using a vertex-based model calibrated to reproduce lateral root primordia emergence, they examine the relation to catenary curves made by hanging chains. This shows that a similar balances of forces are observed in both cases, providing a tentative explanation for the similarity of RAM geometry and catenary curves.

The vertex model is somewhat simplistic, but overall agreement between the models and lateral root primordia outlines in the puchi-1 and the aur1 aur2 mutant help support the idea that this model is sufficient for this study.
Related simulations also help us understand how cell-division and growth contribute to the emergence of a catenary-like form.

Altogether, I feel the study provides valuable insights into RAM development by identifying a conserved feature of root geometry and identifying its developmental origin.

Comments for the author

The idea that the catenary curve is a mechanical consequence of RAM development is reasonably supported. Whether this ensures their mechanical stability still seems unclear to me (e.g. as stated on Lines 51, 104-105). This seems to be a problem for future studies, as the authors seem to indicate on Lines 401-402. I think claims regarding mechanical stability should be rephrased.

The presented results help support the use of the vertex-based model of LRP development, nonetheless how several aspects of the model correspond to the actual mechanics of cell growth merits discussion. In the described model the target-length of each wall appears to be zero (equation on Line 525) and growth is implemented by increasing the target area of cells (Line 559).

© 2021. Published by The Company of Biologists under the terms of the Creative Commons Attribution License (https://creativecommons.org/licenses/by/4.0/).

How this is related to the relatively standard Lockhart model, which assumes that turgor pressure and progressive yielding of the cell walls drives growth, is unclear. As the nature of many arguments is based on a mechanical interpretation of the model, I believe these aspects should be better justified.

On a related note, further examination of these findings using more mechanically faithful models is an important direction for future work (e.g. Bassel et al.). This should be discussed in the manuscript.

Minor comments:

The concept of isometric scalability is unlikely to be well known to most readers, could the authors provide a more intuitive explanation at the first introduction (geometrically similar is a bit vague).

Lines 170-172: Given the small number of species examined, I'm not sure if the absence of a correlation is meaningful. Also, how was this correlation tested?

Line 524: Should F_function be F_tension?

Lines 542-543: “apical end were set rather freely on a giant imaginal cell” is hard to understand, please rephrase.

First revision

Author response to reviewers' comments

>*Reviewer 1 Advance summary and potential significance to field The shape of the plant root tip has been previously reported to match that of an ellipse. In this work the authors report that this structure's curvature also matches a catenary arch, representing the strongest curved structure. These geometric matches are reported for a series of species.*
We thank the reviewer for valuable comments on our manuscript. We revised our manuscript according to the reviewer's advice.

>*Reviewer 1 Comments for the author The implication of the catenary arch is that the shape of the root is mechanically optimized to penetrate soil impedance. The authors proceed to generate models describing the generative processes leading to this curve. The PUCHI and AURORA KINASE mutants are shown to have different LRP shapes, and a plausible mechanism underpinning these geometric differences is proposed. This represents an interesting and well-presented manuscript for which I have no major comments or objections, with the exception of the functional implications of the thesis. If the catenary curve is indeed a mechanically optimised shape in the context of roots penetrating soil, this remains to be demonstrated. The authors have the necessary genetic materials in the form of the wild-type, PUCHI and AURORA mutants to explore the mechanical properties each of these root shapes exhibit in the face of mechanical impedance. It remains important to demonstrate this point as the vector forces applied to an arch on a bridge are different to that of a drill bit penetrating a material. The latter example more closely represents the process of roots entering into soil. The addition of these in vivo mechanical experiments would significantly enhance the conclusions of this manuscript.*
We thank the reviewer for for the constructive comments to help improving our manuscript . We agree with you to the importance of experimenarlly demonstrating that "the catenary curve is a mechanically optimised shape in the context of root penetrating into soil". Although LRP oulines of our selected mutants (*aur1 aur2* and *puchi-1*) deviate from a catenary curve, the shape became similar to a catenary shape in mature LRs and PRs, probably due to the establishment of the meristem organization and tissue growth rules intrinsic to the root meristem. Thus, "genetic materials" to be compared with wild-type for penetration ability is not immediately available, while we deeply appreciate the reviewer's suggestion. In addition, *in planta* measurement of mechanical force during root penetration is extremely challenging.

© 2021. Published by The Company of Biologists under the terms of the Creative Commons Attribution License (https://creativecommons.org/licenses/by/4.0/).

As an alternative approach, we calculated force distribution on growing LRP outlines *in silico*. We found that the vertical force was spatially uniformly loaded along a catenary-curved outline (Fig. 4I), whereas it was less uniformly loaded on outlines deviating from a catenary curve (i.e. the shallow gradient model and the randomized division model) and localized at some areas (revised supporting Figure S8F-G). This theoretical result indicates that the mechanical force produced by tissue growth is spatially homogenized along the surface of catenary-curved root tips, and this notion may be extended further to support the idea that the force produced by the interaction between growing root and the soil could be also equally loaded on the entire surface of the catenary-shaped root tips. We added new data as Figure S8F and S8G for the force distribution *in silico* and described it in the Results (Line 262-263 and 289-290). Corresponding statements on the possible future studies in the Discussion have also been revised (Line 411-420).

>*Minor points relate to the abstract where the use of mutants and the computational simulations merit mentioning.*
We have revised the Abstract to reflect the reviewer's suggestion (Line 41-46).

>*Reviewer 2 Advance summary and potential significance to field This study provides interesting insights into the developmental constraints shaping the geometry of Root Apical Meristems (RAMs). It shows that RAMs in Arabidopsis and 9 other species can be well approximated by a catenary curve. This allows root tip geometry to be characterized by a single shape parameter alpha, which is roughly the inverse of curvature. The ability to capture overall wild-type RAM geometry as a single interpretable shape parameter is noteworthy and seems useful for future quantitative studies, as is the finding that the various RAMs analyzed exhibit isometric scaling. Although other functions approximate RAMs similarly (e.g. ellipses), catenary curves have noteworthy mechanical properties. Using a vertex-based model calibrated to reproduce lateral root primordia emergence, they examine the relation to catenary curves made by hanging chains. This shows that a similar balances of forces are observed in both cases, providing a tentative explanation for the similarity of RAM geometry and catenary curves. The vertex model is somewhat simplistic, but overall agreement between the models and lateral root primordia outlines in the puchi-1 and the aur1 aur2 mutant help support the idea that this model is sufficient for this study. Related simulations also help us understand how cell-division and growth contribute to the emergence of a catenary-like form. Altogether, I feel the study provides valuable insights into RAM development by identifying a conserved feature of root geometry and identifying its developmental origin.*
We appreciate the reviewer's favorable comments on our manuscript. Please see below our response to the specific comments.

>*Reviewer 2 Comments for the author The idea that the catenary curve is a mechanical consequence of RAM development is reasonably supported. Whether this ensures their mechanical stability still seems unclear to me (e.g. as stated on Lines 51, 104-105). This seems to be a problem for future studies, as the authors seem to indicate on Lines 401-402. I think claims regarding mechanical stability should be rephrased.*
We agreed with the reviewer in this point. We now deleted the phrase "mechanical stability" from the Abstract, and revised the Discussion by separating the descriptions of the mechanical consequence from LRP development and the resulting mechanical property that possiblly facilitates root penetration to soil (Line 415-420).

>*The presented results help support the use of the vertex-based model of LRP development, nonetheless how several aspects of the model correspond to the actual mechanics of cell growth merits discussion. In the described model the target-length of each wall appears to be zero (equation on Line 525) and growth is implemented by increasing the target area of cells (Line 559). How this is related to the relatively standard Lockhart model, which assumes that turgor pressure and progressive yielding of the cell walls drives growth, is unclear. As the nature of many arguments is based on a mechanical interpretation of the model, I believe these aspects should be better justified. On a related note, further examination of these findings using more mechanically faithful models is an important direction for future work (e.g. Bassel et al.). This should be discussed in the manuscript.*
We thank the reviewer for the constructive suggestion. In response to this comment, we added a description comparing the standard Lockhart model and our vertex-based model, and discussed how our model can be improved in future to fit the mechanics of actual cell growth (Line 554-558 in

© 2021. Published by The Company of Biologists under the terms of the Creative Commons Attribution License (https://creativecommons.org/licenses/by/4.0/).

Material and Method, and Line 404-407 in Discussion). As pointed out by the reviewer, the standard Lockhart model assumes that plastic deformation of cells during growth is represented by multiplication of excess turgor pressure over yield stress and cell wall extensibility (Lockhart 1965). In our vertex-based model, the plastic deformation was formulated by irreversible increase in target cell areas, again as pointed out by the reviewer. While our vertex model simulates the tissue growth rules to shape the root tip, and the force distribution on its outline, formulation of plastic deformation would improve the model to be more consistent with the Lockhart model. Plastic deformation caused by cell wall extensibility can be formulated by increasing the preferred cell edge length (the second term in Eqn. 14) instead of increasing the preferred area. The plastic deformation by turgor pressure can be reformulated by multiplying turgor pressure with cell area (the first term in Eqn. 14). In addition to the plastic deformation, plant cells also deform reversibly, exhibiting elastic deformation. Synthetic nature of the two types of deformation (plastic and elastic (elasto-plastic)) can be incorporated into the present vertex-based model to follow the Lockhart-Ortega model (Ortega 1985), by replacing the second term in Eqn. 14 with squared-difference between the cell edge length and the target length. These improvements of the model will help clarifying development constraints derived from cell wall extensibility and turgor pressure of individual cells that we demonstrated to underlie the formation of catenary-shaped root tips.

>*Minor comments: The concept of isometric scalability is unlikely to be well known to mostreaders, could the authors provide a more intuitive explanation at the first introduction (geometrically similar is a bit vague).*
Scaling nature of biological shapes has been described in the Discussion (Line 327-339). Following the reviewer's suggestion, we added an intuitive explanation for the isometric scalability to the Introduction (Line 96-98).

>*Lines 170-172: Given the small number of species examined, I'm not sure if the absence of a correlation is meaningful. Also, how was this correlation tested?*
We did not perform statistical tests of correlation, and thus removed the corresponding sentence from the text.

>*Line 524: Should F_function be F_tension?*
Following the reviewer's suggestion, we revised the expression to F_tension (Line 545).

>*Lines 542-543: "apical end were set rather freely on a giant imaginal cell" is hard to understand, please rephrase.*
Following the reviewer's suggestions, we revised the correspoding sentence to "apical end were displaceable in any direction. Below 40 μm of the LRP height, those at the apical end are adjacent to an imaginal cell to mimic the overlaying parental cells" (Line 569-570).

Second decision letter

MS ID#: DEVELOP/2020/196253

MS TITLE: Tissue growth constrains root organ outlines into an isometrically scalable shape

AUTHORS: Motohiro Fujiwara, Tatsuaki Goh, Satoru Tsugawa, Keiji Nakajima, Hidehiro Fukaki, and Koichi Fujimoto
ARTICLE TYPE: Research Article

I am happy to tell you that your manuscript has been accepted for publication in Development, pending our standard ethics checks.

© 2021. Published by The Company of Biologists under the terms of the Creative Commons Attribution License (https://creativecommons.org/licenses/by/4.0/).

Reviewer 1

Advance summary and potential significance to field

The authors have identified an interesting link between root structure and function.

Comments for the author

The authors have made a reasonable attempt to address my comments. The inclusion of these simulations represents a link between structure and putative function. Performing these mechanical experiments in vivo is indeed a complex proposition.

Reviewer 2

Advance summary and potential significance to field

As with the original submission, I still feel that the study provides valuable insights into RAM development by identifying a conserved feature of root geometry and identifying its developmental origin.

Comments for the author

I thank the author's for their careful revision of the manuscript to incorporate my previous comments. I believe the manuscript is now suitable for publication.

© 2021. Published by The Company of Biologists under the terms of the Creative Commons Attribution License (https://creativecommons.org/licenses/by/4.0/).
